# Four methods of brain pattern analyses of fMRI signals associated with wrist extension versus wrist flexion studied for potential use in future motor learning BCI

**DOI:** 10.1371/journal.pone.0254338

**Published:** 2021-08-17

**Authors:** Aniruddh Ravindran, Jake D. Rieke, Jose Daniel Alcantara Zapata, Keith D. White, Avi Matarasso, M. Minhal Yusufali, Mohit Rana, Aysegul Gunduz, Mo Modarres, Ranganatha Sitaram, Janis J. Daly

**Affiliations:** 1 J. Pruitt Family Department of Biomedical Engineering, College of Engineering, University of Florida, Gainesville, Florida, United States of America; 2 Brain Rehabilitation Research Center, Malcom Randall VA Medical Center, Gainesville, Florida, United States of America; 3 Department of Psychology, College of Public Health and Health Professions, University of Florida, Gainesville, Florida, United States of America; 4 Department of Chemical Engineering, College of Engineering, University of Florida, Gainesville, Florida, United States of America; 5 Laboratory for Brain-Machine Interfaces and Neuromodulation, Pontificia Universidad Católica de Chile, Santiago, Chile; 6 Institute for Biological and Medical Engineering, Pontificia Universidad Católica de Chile, Santiago, Chile; 7 Department of Psychiatry and Division of Neuroscience, Pontificia Universidad Católica de Chile, Santiago, Chile; 8 Department of Neurology, College of Medicine, University of Florida, Gainesville, United States of America; Columbia University, UNITED STATES

## Abstract

**Objective:**

In stroke survivors, a treatment-resistant problem is inability to volitionally differentiate upper limb wrist extension versus flexion. When one intends to extend the wrist, the opposite occurs, wrist flexion, rendering the limb non-functional. Conventional therapeutic approaches have had limited success in achieving functional recovery of patients with chronic and severe upper extremity impairments. Functional magnetic resonance imaging (fMRI) neurofeedback is an emerging strategy that has shown potential for stroke rehabilitation. There is a lack of information regarding unique blood-oxygenation-level dependent (BOLD) cortical activations uniquely controlling execution of wrist extension versus uniquely controlling wrist flexion. Therefore, a first step in providing accurate neural feedback and training to the stroke survivor is to determine the feasibility of classifying (or differentiating) brain activity uniquely associated with wrist extension from that of wrist flexion, first in healthy adults.

**Approach:**

We studied brain signal of 10 healthy adults, who performed wrist extension and wrist flexion during fMRI data acquisition. We selected four types of analyses to study the feasibility of differentiating brain signal driving wrist extension versus wrist flexion, as follows: 1) general linear model (GLM) analysis; 2) support vector machine (SVM) classification; 3) ‘Winner Take All’; and 4) Relative Dominance.

**Results:**

With these four methods and our data, we found that few voxels were uniquely active during either wrist extension or wrist flexion. SVM resulted in only minimal classification accuracies. There was no significant difference in activation magnitude between wrist extension versus flexion; however, clusters of voxels showed extension signal > flexion signal and other clusters vice versa. Spatial patterns of activation differed among subjects.

**Significance:**

We encountered a number of obstacles to obtaining clear group results in healthy adults. These obstacles included the following: high variability across healthy adults in all measures studied; close proximity of uniquely active voxels to voxels that were common to both the extension and flexion movements; in general, higher magnitude of signal for the voxels common to both movements versus the magnitude of any given uniquely active voxel for one type of movement. Our results indicate that greater precision in imaging will be required to develop a truly effective method for differentiating wrist extension versus wrist flexion from fMRI data.

## Introduction

Wrist movement impairment is common in chronic stroke (6+ months post-stroke), even after standard therapy is complete. Tasks of daily living require coordinated wrist and forearm joint movements of flexion-extension, radial-ulnar deviation, and forearm pronation-supination [1]. In particular, wrist extension is critical to achieving a functional position of the hand and fingers for grasp and object manipulation during occupational and recreational activities [2].

Conventional and emerging therapies have had limited success in functional recovery for patients with chronic or severe motor impairment [3,4]. Our previous work was promising but required long-duration treatment [5]. Some have studied brain-computer interfaces (BCIs) to provide neural feedback to stroke survivors attempting to re-learn coordination [6,7]. BCIs for rehabilitation are designed to drive the reorganization of neural circuits by providing feedback contingent upon brain activity evoked during motor execution or motor imagery [8,9].

Electroencephalography (EEG)-based BCIs show some promise and mixed results [10–16]. One limitation of EEG-based BCIs is the lack of spatial precision in identifying and targeting brain regions for feedback. In contrast, functional MRI provides higher spatial resolution, potentially enabling a BCI to better identify specific brain regions to employ for motor re-learning strategies [17]. Though no studies have published recovery of motor control, there is evidence that fMRI neurofeedback has enabled participants to volitionally regulate their brain signal from the primary motor cortex [18,19] and pre-motor cortex [20,21]. Stroke survivors have shown acquired self-modulation of ventral premotor cortex [22] and motor cortico-thalamic communication [23]. These studies are not examples of fine motor learning applications. Nevertheless, they present initial quantified evidence of participants modifying their brain signal in motor-related regions through neural feedback. Based on these initial studies, it is reasonable to consider that MRI-based BCIs could potentially contribute to improved motor control. For example, our group studied feasibility of using real time fMRI as the basis of a BCI system for motor learning after stroke [24]. Though promising, these studies, including our own, have not solved a number of problems inherent in a brain neural feedback system designed to improve dyscoordinated movement.

One of those problems is the current treatment-resistant problem in which undesirable co-contraction of muscles occur which are antagonistic to the desired movement [25] and ineffective activation and contraction of the desired agonist muscles. For example, after stroke, wrist coordination is impaired such that the intention and effort to execute wrist extension is characterized by undesired co-contraction of wrist flexor muscles. This is antagonistic to the intended wrist extension and may result in the opposite movement. Given such motor impairment, we reasoned that an important motor control re-learning goal is to modulate accurately the differential activation of wrist extensor versus wrist flexor muscles, for either the extension or the flexion movement, depending upon the intended task. We further reasoned that to re-learn such modulated brain control, it would be most informative to the stroke survivor to have a ‘window’ into their own brain activations during intended flexion versus extension movements of the wrist joint. Therefore, the purpose of this study was to learn if we could identify differential neural activities distinguishing the simple wrist extension movement from wrist flexion movements, to improve future studies of image-guided BCI feedback therapy. Thus, this critical information could serve as the basis for a future neural feedback motor learning system.

Others have reported the ability to differentiate brain signals of differing movements. With limited success, others have attempted to utilize electroencephalography (EEG) to differentiate between both real and imaginary movements of the right wrist [26,27]. Researchers have used functional magnetic resonance imaging (fMRI) to discriminate between different types of motor tasks as follows: right-hand actions, according to whether these responses were evoked using motor imagery or motor execution [28,29]; prediction of handedness using functional connectivity of primary motor cortex and dorsal premotor cortex [30]; intrinsic versus extrinsic action coordinate frames, utilizing isometric wrist extension-flexion [31]. There remains a gap in the literature in which there has been no establishment of brain signal pattern that can differentiate simple volitional extension movement of the wrist versus simple volitional flexion movement of the same joint, a gap that the present study attempts to address in part. This differentiation of wrist extension brain signal from wrist flexion brain signal is critical in the future application of BCI systems to the problem of wrist dyscoordination after stroke.

Therefore, given the potential promise of that information for the stroke survivor attempting to re-learn wrist coordination and the fact that it is not known how the brain drives perfectly modulated wrist extension and wrist flexion in normal adults, we conducted necessary initial exploratory work in healthy adults to characterize differential brain signal for wrist extension versus wrist flexion. For this, we selected four models of analysis, each with different advantages, mainly used in fMRI, and looked to be promising for the future purpose. These four models were: the univariate analysis, general linear model; multivariate classification analysis, support vector machine; the winner take all voxel-wise t-statistics; and the voxel-wise relative percent signal change for action preference.

The purpose of this paper is to describe the methods we used and the results that we obtained so that others will realize that even more refined approaches are needed to obtain more definitive conclusions. Using these four models to analyze our group and individual data, we have gained knowledge about and are presenting the steps required to improve the design of future studies and increase the likelihood of a future successful outcome.

## Methods

### Study design

We acquired functional MRI data from a cohort of healthy participants who performed right wrist flexion and right wrist extension movements. We applied four different types of off-line analyses to explore the feasibility of differentiating brain activation patterns for wrist extension versus wrist extension.

### Participants

We enrolled 11 healthy individuals (7 males and 4 females; average age 43.8 ± 22.9 years). All participants were right-handed except one male. Participants gave written informed consent prior to the study in accordance with the Declaration of Helsinki. The study was approved by University of Florida’s human subjects’ protection oversight board.

One female participant (C07) was excluded from further analysis based on an excessive number of artifacts from head motions. That is, each run had greater than or equal to 46/116 images classified as containing artifacts, as follows: run 1 = 46/116; run 2 = 60/116; run 3 = 58/116; run 4 = 56/116. The resulting sample size was ten participants.

### Procedures and analysis

#### Behavioral protocol

**Fig 1:**
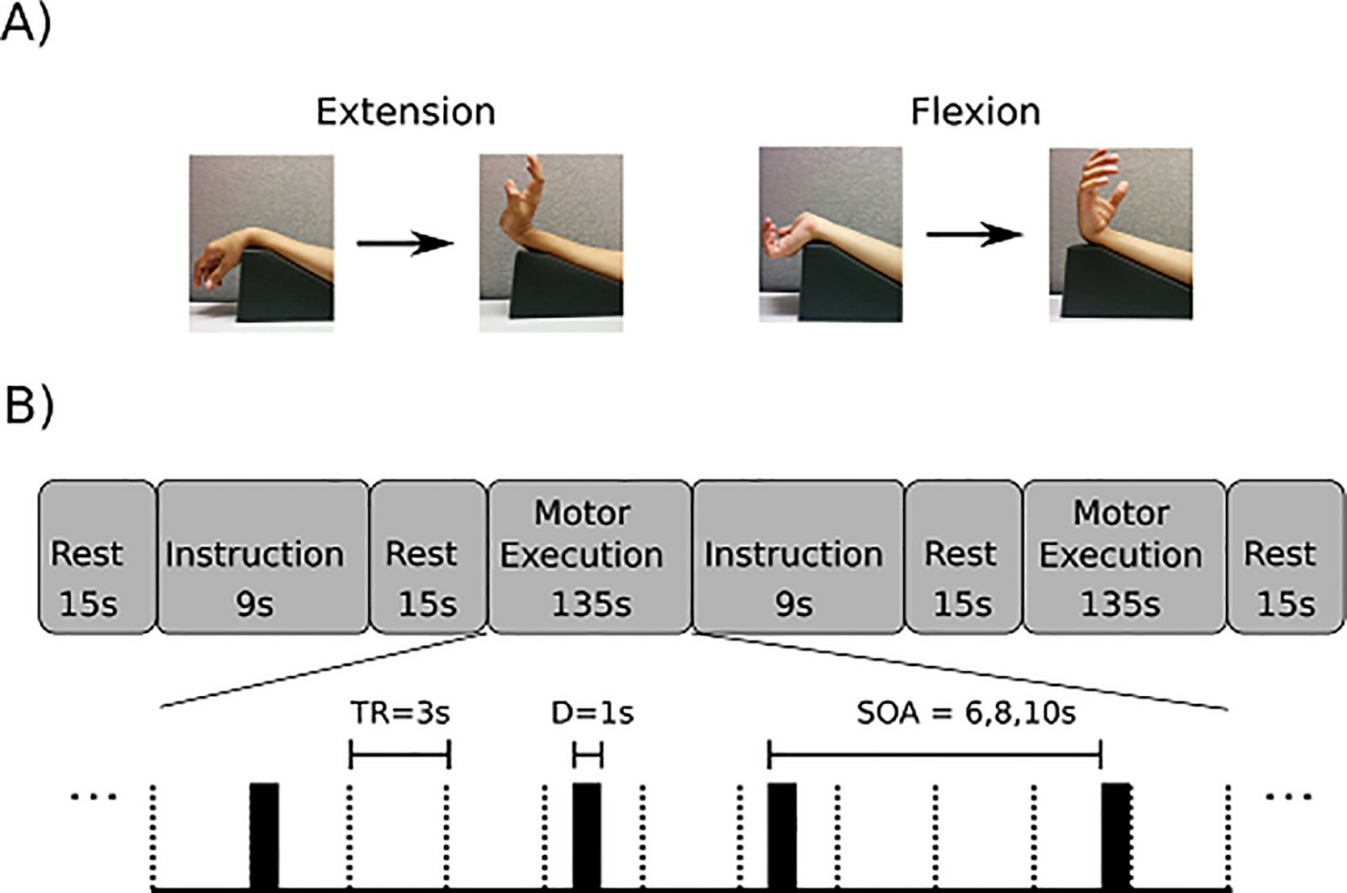
Study design. Panel A. Arm positions used for wrist extension and wrist flexion movements. Right wrist extension executed against gravity: The forearm was pronated and a foam wedge positioned the elbow flexed 30 deg. Right wrist flexion against gravity: The forearm was supinated, with the elbow positioned in 30 degrees of elbow flexion. Each repetition of the motor task was cued by the word “Go” appearing on the screen, and the movement end-point (e.g., full wrist extension) was instructed to be held for 1 sec, after which time the instruction on the screen changed to a fixation figure, and the participant lowered the hand to the start position. Panel B. Blocked data acquisition design, with each movement cued within a given block, for one run of the wrist movement task. The upper part of panel B shows the sequence of rest, instruction, and execution periods. The lower part of panel B shows a detailed subsample of a motor execution series on an expanded time scale. In this subsample, movement events are cued at pseudo-random intervals.

The fMRI motor task protocol consisted of a blocked design, and further, each movement was cued within a given block (that is, each of the cued movements was considered an ‘event’ within the given block. This design is illustrated in Fig 1. We will refer to this design as a mixed blocked and event-related design. The visual cueing was provided for each movement at pseudo-random intervals. A first type of task block was defined as a series of 15 flexion movements. A second type of task block was defined as a series of 15 extension movements. A ‘run’ consisted of two task blocks, with one block containing a series of 15 repetitions of extension movements and a separate task block containing a series of 15 repetitions of flexion movements, with a 15 s rest period prior to and after each block. At least four runs were completed by each participant. Visual instructions were displayed at the beginning of a series of either wrist extensions or wrist flexions. During this instruction period, the participant adopted the forearm posture (pronation or supination) required for the forthcoming movement series. The motor task was simple, active wrist extension or flexion, performed against gravity. We selected this motor task for the study of brain control because it is the task which the stroke survivor must master before progressing to more complex or more forceful movements. Without the mastery of simple wrist movement (against only gravity), the stroke survivor is not able to progress to more functionally relevant motor learning training.

We varied stimulus onset (stimulus onset asynchrony (SOA)) between consecutive movement cues during the motor execution period (6, 8, or 10 seconds), in order to improve characterization of the blood-oxygenation-level dependent (BOLD) response and to minimize habituation and anticipatory effects (Fig 1 subpanel B).

#### Imaging protocol

We acquired structural and functional MRI data using a 3 Tesla Philips Achieva scanner. T1-weighted structural images were acquired [MPRAGE sequence, TE = 3.2 ms; TR = 7.0 ms; flip angle (deg) = 8; matrix size = 240*240; 176 sagittal slices, 1 mm isotropic voxels]. Functional T2*-weighted echo-planar images were acquired [TE = 30 ms; TR = 3000 ms; SENSE factor = 2; flip angle (deg) = 90; matrix size = 80*80; 43 axial slices; 3 mm isotropic voxels; 4 ‘dummy’ + 116 acquired images per run (6 min)]. Dummy images at the beginning of each run allowed the magnetization to reach steady-state and were subsequently discarded.

Regions of interest (ROI) for between-subject comparisons were obtained in MNI common coordinates from the Human Motor Area Template (HMAT). ROI’s included the following: each hemisphere’s primary motor area (M1), primary somatosensory area (S1), dorsal premotor cortex (PMd) and ventral premotor cortex (PMv), supplementary motor area (SMA) and pre-supplementary motor area (pre-SMA) [32]. We used locations and boundaries of these regions that were previously established by others and reported in a meta-analysis utilizing activation likelihood estimation (ALE) from 126 papers that had used motor tasks and reported activation foci from any combination of the six motor-related cortices [32]. These regions were investigated based on their putative functions [33], and have been reported on extensively in the literature. Additionally, activation of contralateral primary motor and ventral premotor regions have been associated with wrist extension-flexion movements [31].

In addition, for within-subject analyses we investigated Brodmann areas (BA) BA3 (primary somatosensory cortex), BA4 (primary motor cortex), and BA6 (premotor and supplementary motor cortex) using masks derived from participant-specific cortical parcellations generated by FreeSurfer [34]. The participant-specific BA4 masks were further subdivided by identifying each hemisphere’s “hand knob”, using the criteria of Yousry, et al. [35]. The purpose of subdividing BA4 in this fashion was to better localize fMRI activations within the somatotopic organization of primary motor cortices [36], inasmuch as activations related to wrist-activity are often found within or near the margin of the “hand knob”.

We applied four different signal analysis methods to assess their usefulness in differentiating wrist extension from wrist flexion: 1) general linear model (GLM) analysis; 2) support vector machine (SVM) classification; 3) ‘Winner Take All’; and 4) Relative Dominance.

*Univariate analysis; general linear model*, *first type of analysis used*. Statistical Parametric Mapping 12 (SPM12; Wellcome Trust Centre for Neuroimaging, UCL, UK) running on MATLAB R2015a (MathWorks Inc., Natick, MA) was used to preprocess and analyze functional MRI data. The functional images were realigned, slice-time corrected, and co-registered to the participant’s T1-weighted structural image. The Artifact Detection Tool (https://www.nitrc.org/projects/artifact_detect/) was used to identify global mean signal outliers and motion artifacts. Global mean signal outliers were defined as images having standard scores exceeding Z = 3. Motion artifacts were defined as those images that exceeded thresholds of 0.9 mm framewise translational or 0.01 radian rotational displacement [37]. Hereafter, we shall refer to the combination of global mean signal outliers and motion artifacts as artifacts.

The number of motor trials available for analysis for each participant, before artifact rejection, was at least 120 (60 for wrist flexion and 60 for wrist extension) except for one participant who had 90 trials (45 for wrist flexion and 45 for wrist extension) due to a corrupted data file during one run.

Spatial normalization was performed to bring the functional images into Montreal Neurological Institute (MNI; voxel-size 3mm x 3mm x 3mm) space for group analysis. A Gaussian filter of 6mm full-width half maximum was used for spatial smoothing [38] to mitigate remaining individual variations. Wrist extension-flexion onsets were convolved with SPM’s canonical HRF basis set to create regressors for the two conditions. Confound regressors included six head motion parameters generated from the rigid-body transformation of the realignment procedure and the confound matrix generated by the Artifact Detection Tool, which includes separate regressors for each timepoint that is considered an artifact. A high-pass filter was implemented using the discrete cosine transform (DCT) with a default cut-off of 128 seconds. Based on the general linear model (GLM), we derived t-statistic maps for the following contrasts: wrist flexion versus rest, wrist extension versus rest, and wrist extension versus wrist flexion. Group-level images were assessed for cluster-wise significance using a forming threshold of p < 0.001 uncorrected and p < 0.05 family-wise-error (FWE) corrected critical cluster size.

#### Multivariate classification analysis; Support Vector Machine (SVM) classification; second type of analysis used

In contrast to the GLM univariate approach, multi-voxel pattern analysis considers data from multiple voxels, considering spatial patterns of activity. We used both the PRoNTo software package [39] and the MANAS (v4) software toolbox [40,41] for performing offline classification of fMRI signals. We report only the findings from the MANAS software analysis because Pronto results were similar. Support Vector Machine (SVM) was used as the classifier [42], as it has been shown to be a robust method of classification when applied to fMRI data [43].

We report classification results using acquisition-space (realigned and slice-time corrected) as well as spatially normalized (realigned, slice-time corrected, normalized to MNI coordinates) BOLD fMRI timeseries. We accounted for the delay and dispersion of the hemodynamic response. We specified a parameter to delay signal onsets to account for the time-to-peak of the hemodynamic response after stimulus presentation. This delay was set to 6s [39]. Preprocessing consisted of artifact rejection as described for the univariate analysis as well as high-pass filtering to remove signal drift and low frequency noise from the fMRI time series before subsequent feature selection and classification steps. High-pass filtering was performed using a non-linear filter as proposed by Marchini and Ripley [44] and implemented in FSL [45]. This approach fits and removes a linear regression (Gaussian-weighted line of fixed width) and has been found robust in trend removal [46]. Given the randomized SOA, we set the width of this Gaussian (full-width half-maximum) as 80 TRs, which corresponds roughly to 1.5 times the length of one motor action block. Trend removal using the same DCT approach as with univariate analysis above was not available in the MANAS software package. However, the periods of the two detrending methods are similar (128-s cut-off DCT for univariate and 240-s (80 TRs, 3-s TR) full half-height width Gaussian filter for multivariate), and both attenuate frequencies (drifts) below about 0.01 Hz. Neuronal activation (BOLD) frequencies are an order of magnitude higher, theoretically centered around the reciprocal of the average interval (8 s) between consecutive motor cues, or about 0.125 Hz. Thus, the small differences in detrending method cut-offs are inconsequential to BOLD signal detection;

The selection of features, i.e. dependent variables, consisted of (1) inclusive masking of voxels in the region(s) of interest followed by (2) extracting features (BOLD signal values) corresponding to wrist extension or wrist flexion and (3) ranking of features determined by mutual information. A threshold based on the maximum number of features was then applied to return the most relevant subset of voxels for classification. The maximum number of features was chosen to be 2000.

Model specification and evaluation was performed within participant. Performance of classification models was evaluated using 10-fold cross-validation to determine accuracy, sensitivity and specificity. Ten-fold cross-validation is a technique to evaluate a classification model by partitioning the data into a training set to train the model, and an independent test set to evaluate the model.

#### Functional maps of wrist action preference

*“Winner take all” based on t-statistic; third type of analysis used*. To identify the voxel-wise dominance for either wrist extension or flexion, we employed a “winner take all” approach [47]. Only if a given voxel survived a threshold of p < 0.001 (uncorrected) in the ‘wrist flexion versus rest’ or ‘wrist extension versus rest’ contrast was it considered for WTA analysis such that for each suprathreshold activated voxel the winning movement corresponded to the movement with the larger t statistic. Using this criterion, even if flexion and extension returned similar t values, one was always larger than the other, and a winning movement could be defined. Each voxel was assigned a label based on the winning movement. Label maps were created in MNI coordinate for both extension and flexion actions for each participant.

Spatial probability maps for both extension and flexion were based on a voxel-wise sum of the WTA label across subjects. Frequency estimates of the between-subjects probability that each voxel was dominant for a given condition were constructed in the MNI coordinate system by counting the number of times that a label occurred at a given voxel in this common space across all participants. Therefore, each activated voxel was assigned a value ranging from 1 to 10 (the total number of participants), which represents the between-subject probability that the voxel was dominant for a given condition. In addition to these spatial probability maps, the Jaccard similarity coefficient, which represents the degree of overlap of label sets, was used to assess the similarity of maps across participants. Jaccard similarity is the ratio of intersection to union.

*Relative dominance based on percent signal change; fourth type of analysis used*. In a study by Huber et. al., the difference in percent signal change between experimental conditions was used to determine each voxel’s relative dominance for one condition versus the other [48]. After all images were realigned and slice-time corrected using SPM12, linear de-trending was performed and the difference in percent signal change between flexion and extension was calculated on a per voxel basis. Baseline values were calculated using median values of the preceding and the subsequent rest periods, which medians were then averaged. This was done to minimize effects of residual drift. Within-session averages were calculated voxel-wise for each participant.

Run-to-run reproducibility within sessions was assessed using a technique similar to the WTA approach. Each voxel was assigned a label based on the sign of the difference between extension and flexion in terms of percent signal change. Those voxels exhibiting greater percent signal change during the extension condition were assigned a value of 1, and 0 was assigned otherwise. Voxel-wise sums across runs were compared to binomial distributions representing the null hypothesis of random assignment.

## Results

### Summary of results from the four selected analysis models

**The****Univariate analysis, General Linear Model** was the first group-level analysis model we applied to the problem. We selected this method as the first model because of its demonstrated sensitivity for identifying fMRI activations in a wide breadth of other applications.

This model did not prove useful in differentiating wrist extension from wrist flexion with our data. The disadvantages of this model included a lack of specialization for differentially classifying wrist extension from wrist flexion according to features of spatial and temporal patterns of brain activity. These features were not known in advance, and thus, for this model, they could not be specified.

**Multivariate classification analysis, Support Vector Machine** was the second model we applied to the problem. We selected this machine learning method because of its relatively greater sophistication for classifying activations by considering initially unknown spatial-temporal patterns of activity, the predictive features of which were estimated from training on subsets of the data.

Discriminant analysis with support vectors has different strengths and weaknesses compared to the initial GLM analysis, but this second model did not inform us as desired with our data. This method is traditionally used for group-level analysis. Group level analysis often has the disadvantage that it blurs rather than sharpens fine patterns evident within the individual subjects’ data.

**The Winner Take All** based on equivalent t statistic, was the third model that we applied to the problem. We selected this model because of its capability to select, voxel-wise, the dominance of activation for either wrist extension or wrist flexion, applied to observation of individual subject spatial activation patterns. Winner Take All is a non-linear figure of merit representing the larger of extension-related and flexion-related equivalent t statistics for each voxel.

This model did not uncover a consistent spatial pattern across subjects to differentiate simple wrist extension from wrist flexion with our data. Given the lack of desired result with this model and its statistical operations that were applied to the data, we determined that a more direct approach may have merit. It was reasonable to consider the potential of using simply the acquired BOLD percent signal change as the brain signal measure, without any modification by any further statistical considerations.

**The Percent Signal Change for Action Preference** was the fourth model that we applied to the problem. We selected this model next because it is a direct approach using percent signal change to generate voxel-wise figures of merit representing action preference (wrist extension or flexion). This model also did not uncover a consistent spatial pattern across subjects to differentiate simple wrist extension from wrist flexion with our data.

### Univariate analysis results; first type of analysis

In preparation for group level analysis, we generated and visually inspected activation maps for each participant during wrist extension and wrist flexion. We observed high individual variability in brain activation patterns for both wrist extension and wrist flexion. In individual data, voxels could readily be identified in motor cortices which behaved significantly differently during wrist extension versus rest or during wrist flexion versus rest. On average, flexion-related activation volumes exceeded extension-related activation volumes, but a greater number of voxels were common between these wrist actions. In brain maps for any given individual, these unique voxels were closely adjacent to voxels that were common to wrist extension and flexion, and the uniquely active voxels appeared to be of lesser volume versus the common voxels.

**Fig 2:**
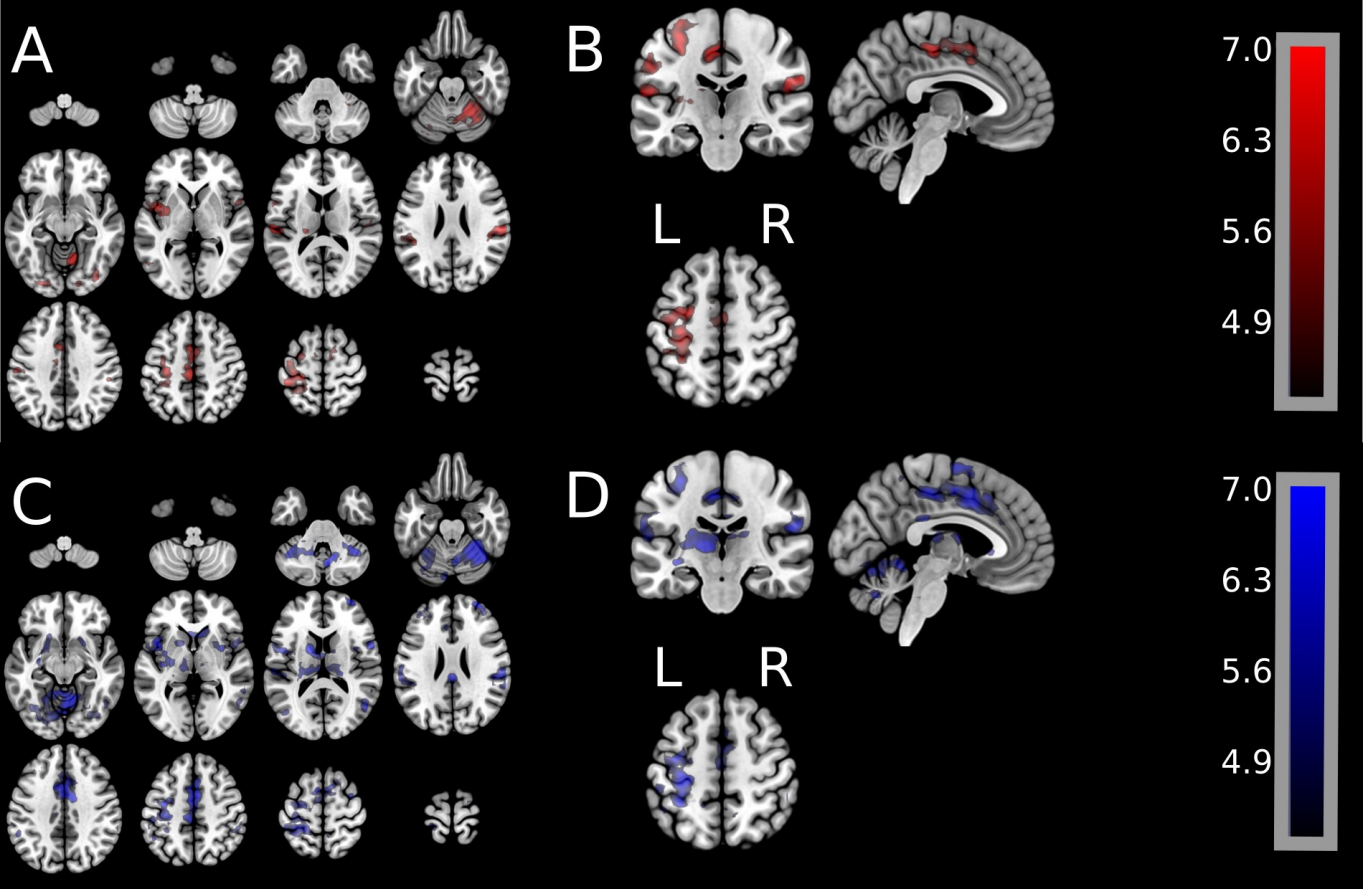
Group-level GLM activation maps for the wrist extension movement and for the wrist flexion movement. Panels A/B show spatial brain activation for wrist extension (extension movement–rest). Panels C/D show spatial brain activation for wrist flexion (flexion movement–rest). Images were assessed for cluster-wise significance using a forming threshold of p < 0.001 uncorrected (p < 0.05 FWE-corrected). Critical cluster sizes were 702 mm^3^ and 945 mm^3^ for wrist extension and wrist flexion, respectively. Color bar for the t-statistics shows the gradient from the lowest to the highest values.

Fig 2 shows group univariate GLM results for all active voxels during either wrist extension or wrist flexion. Fig 2 illustrates the spatial patterns of activation (cluster-wise significance of p < 0.001, FWE p < 0.05). Fig 2 Panels A/B show, for wrist extension, all brain spatial pattern activations. Panels C/D show, for wrist flexion, all activation patterns.

**Fig 3:**
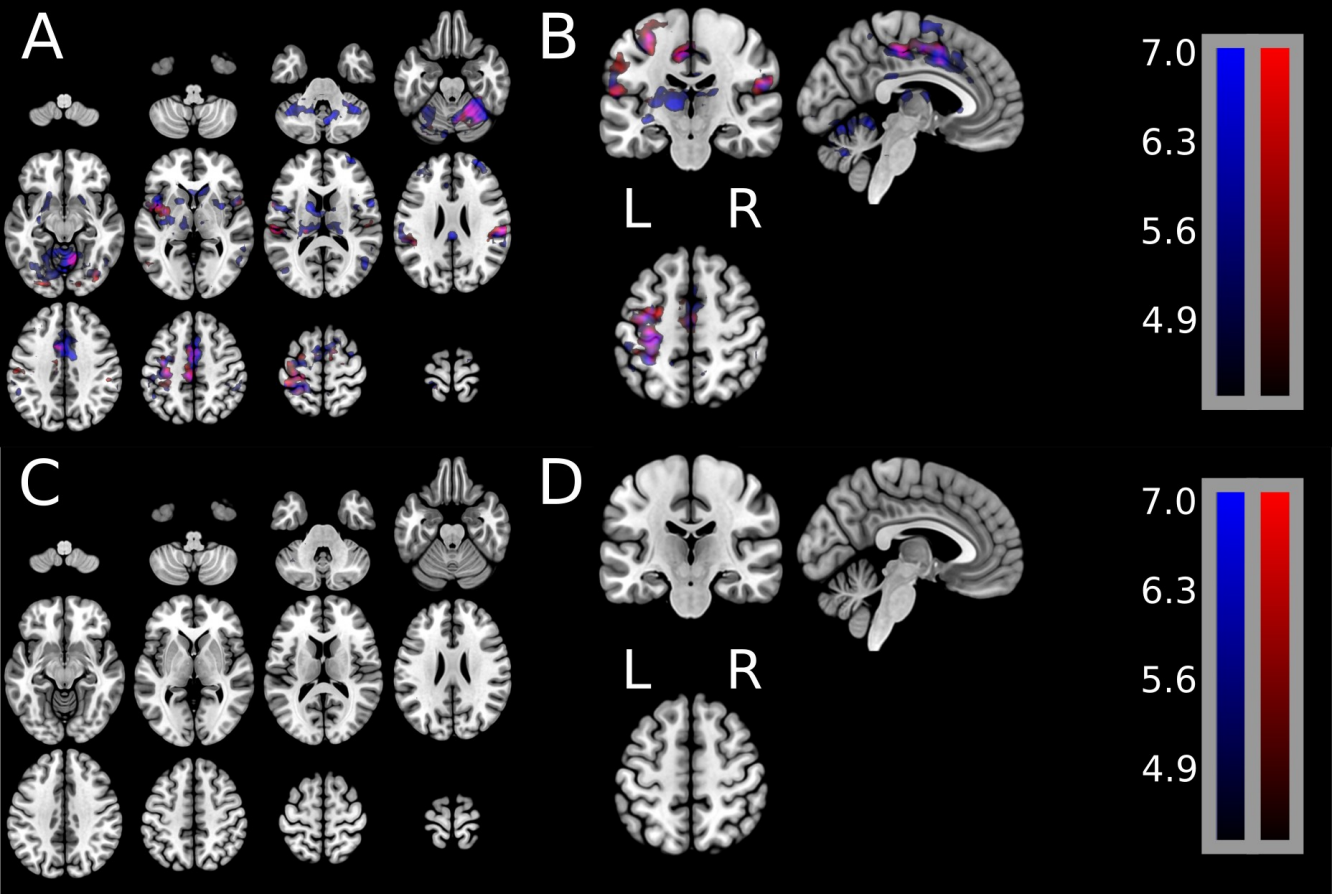
**Group-level GLM activation maps showing unique brain activation spatial patterns during the wrist extension movement (red), unique activation during wrist flexion movement (blue), and shared activation patterns during extension and flexion.** Group-level t-statistic images were created in MNI coordinates for the following contrasts: Wrist extension versus rest, wrist flexion versus rest, and wrist flexion versus wrist extension. **Panels A and B** show one statistical map containing unique brain activations during wrist extension only (red) and unique activations during wrist flexion only (blue), as well as brain activations shared in common during extension and flexion. t-statistic images were assessed for cluster-wise significance using a forming threshold corresponding to p < 0.001. Critical cluster sizes were 702 mm^3^ and 945 mm^3^ for wrist extension and wrist flexion, respectively. **Panels C and D** illustrate the lack of results from calculating the statistically significant difference of greater brain activation during extension versus that during flexion. Fig 3, Panels C/D shows that no regions (no red or no blue regions) survived the statistical significance test.

Fig 3 shows group results of uniquely active brain signal for either extension or flexion as well as the brain signal common during both extension and flexion. Group-level t-statistic images were created in MNI coordinates for the following contrasts: wrist extension versus rest, wrist flexion versus rest, and wrist flexion versus wrist extension. Panels A/B show one statistical map containing unique brain activations during extension only (red) and unique brain activations during flexion only (blue), as well as the brain activations common during extension and flexion (purple). Panels C/D illustrate the lack of results from calculating the statistically significant difference of greater brain activation during extension than during flexion. Fig 3, Panels C/D shows that no regions (no red or no blue regions) survived the statistical significance test.

These contrasts show a high degree of spatial overlap, as illustrated in Fig 3 Panels A/B. Additionally, wrist extension and wrist flexion were directly compared at the group-level (shown in Fig 3 Panels C/D). The group average reveals no significant difference between wrist extension and wrist flexion in either direction: wrist extension > wrist flexion or wrist flexion > wrist extension.

Shown in Table 1, we identified the total volume of activation within each region of interest. The greatest volumes of activation were found within the left primary motor cortex (M1) and primary somatosensory cortex (S1) regions, consistent with actions of the right wrist. The mean volume of activation across participants was greater in the left hemisphere (contralateral to the moving arm) than the ipsilateral hemisphere for most regions with two exceptions: (1) pre-SMA cortex, and (2) ventral premotor cortex evaluated with the wrist flexion versus rest contrast. Regional activation volumes were not compared statistically. We noticed substantial volume variation across participants for both contrasts.

**Table 1:** Volumes (mm^3^) of activated voxels calculated from within-participant univariate analysis in MNI coordinates, averaged across participants.

Brain Regions	Extension (mm^3)^		Flexion (mm^3)^	
	Mean	SD	Mean	SD
M1 R	473	977	1726	3327
M1 L	5859	3980	6888	4717
S1 R	862	1491	1205	1833
S1 L	4909	3606	5025	3715
SMA R	1421	1513	1812	1640
SMA L	2841	2299	3238	2238
preSMA R	1523	1742	1410	1032
preSMA L	1072	1240	1194	1056
PMd R	897	1694	1477	2182
PMd L	2163	1954	2476	1741
PMv R	1129	1724	1637	2108
PMv L	1191	982	1450	1740

Activations were assessed using a cluster forming threshold of p < 0.001 uncorrected, FWE p < 0.05 cluster size correction for wrist extension > rest and wrist flexion > rest contrasts. Brain ROIs are from the Human Motor Area Template. In all ROIs except preSMA in right hemisphere, mean activation volumes for flexion exceeded corresponding mean activation volumes for extension. Transformation to MNI coordinates can distort voxel volumes; however, such distortions are unlikely to differentially affect extension-related versus flexion-related voxels due to their close proximities.

We found that there is a wide variability across healthy adults in terms of their patterns of brain activation, even during the simple tasks of active wrist extension movement and active wrist flexion movement (no resistance, except gravity). Some participants exhibited quite different brain activation patterns during wrist extension versus wrist flexion, with different tissue volumes activated exclusively by one but not the other movement. Thus, in the left primary motor hand knob region, 6/10 participants exhibited volume of activation that was exclusively activated by either extension or flexion; that is, these volumes of activation were not shared across extension and flexion movements. Further, 30% of those subjects had no shared volume of activation at all. In contrast, three participants (3/10) exhibited in the left, hand knob region only ‘shared’ volume of activation during flexion and extension, that is, no unique activations. In the left pre-motor cortex, exclusively activated volume of activation for the extension movement were found in 7/10 participants; and for the flexion movement, exclusive activations were found in 8/10 participants.

### Multivariate classification analysis results; second type of analysis

**Spatially normalized fMRI input.** We report classification accuracy, sensitivity, and specificity associated with discriminating wrist extension and wrist flexion using images realigned, slice-time corrected, and normalized to MNI coordinates. One potential benefit of using spatially normalized images is it enables a participant-independent classifier [41].

Classification performance was calculated per participant using 10-fold cross validation. Accuracy, sensitivity, and specificity were used to assess the performance of classification between wrist flexion and extension. Instances (trials) of wrist flexion and wrist extension executions were randomly partitioned into 10 folds. Training the classifier was conducted on 9 folds, followed by testing (prediction), which was conducted on the remaining 10^th^ fold. Each fold was then evaluated. One advantage of the k-fold cross-validation is that it can account for variability between runs by permitting time series collected in the same run to be split such that one subset of instances is incorporated in the training folds, while a different subset of instances is included in the test fold.

We evaluated all regions incorporated by the Human Motor Area Template (HMAT) including a union of all region masks into a large single mask. Table 2 incorporates the top 3 HMAT regions based on mean accuracy across participants. These regions are the left S1, left ventral premotor cortex (PMv), and left M1 with accuracies of 65.9%, 62.5%, and 61.7%, respectively.

**Table 2:** Top 3 regional accuracies within the Human Motor Area Template (HMAT) and accuracy when using all regions (HMAT union).

Subject		Accuracy (%)		
	Left S1	Left PMv	Left M1	HMAT Union
01	66.0	44.1	43.4	44.9
02	44.3	47.2	53.1	46.8
03	75.7	69.9	73.8	75.3
04	68.3	75.3	71.9	76.7
05	59.3	64.6	70.0	69.4
06	76.4	75.8	70.7	79.8
08	60.2	58.3	48.7	62.3
09	60.3	62.5	58.9	62.8
10	67.4	61.4	62.2	59.9
11	80.8	66.2	64.6	73.9
Mean (SD)	65.9 (10.6)	62.5 (10.6)	61.7 (10.5)	65.2 (12.2)

An SVM was trained to differentiate wrist extension and flexion using spatially normalized images (realigned, slice-time corrected, transformed to MNI space) evaluated using 10-fold cross validation.

**Fig 4:**
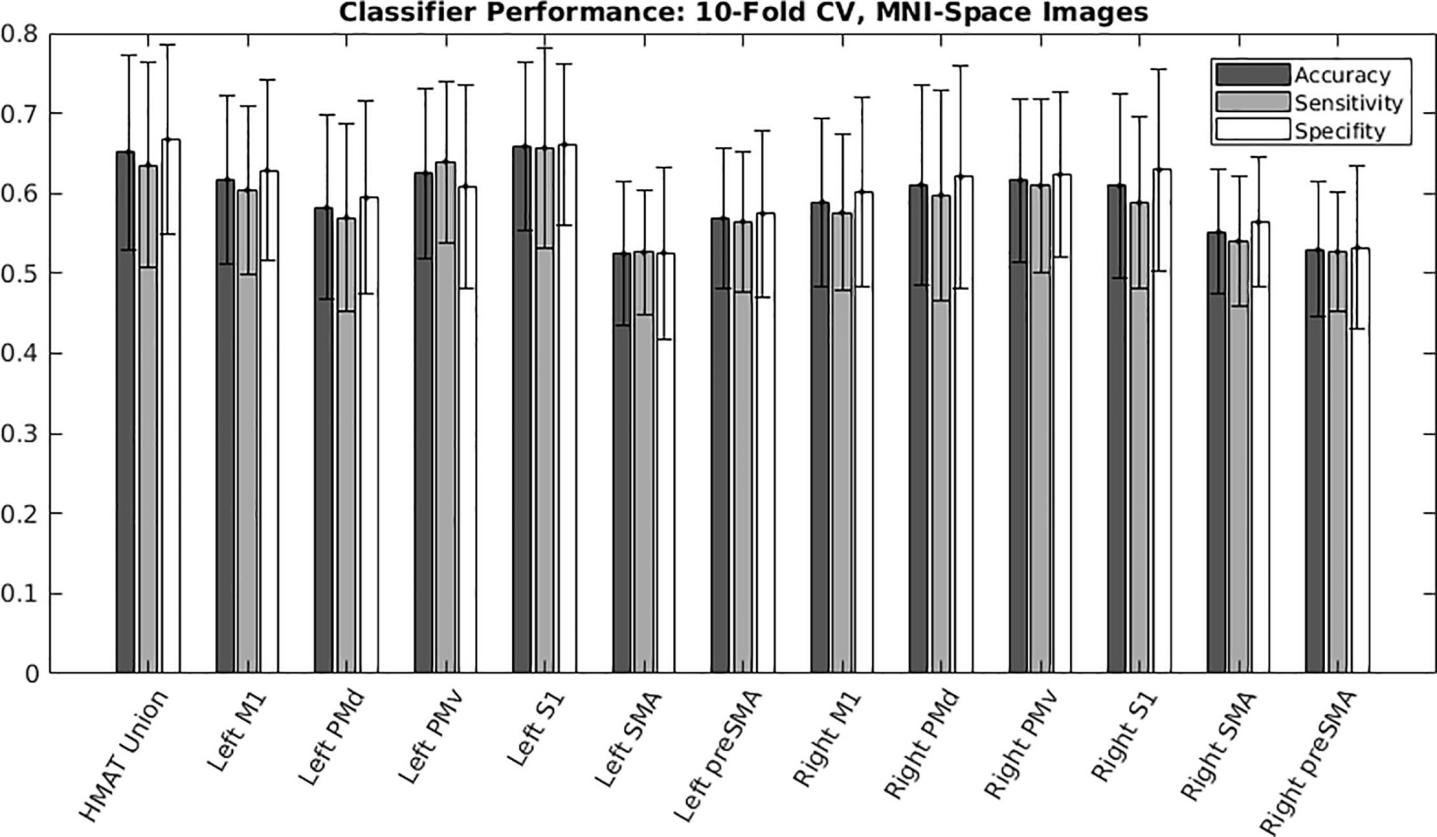
MNI-space images: Regional classifier performance in terms of accuracy, sensitivity, and specificity. We evaluated all regions incorporated by the Human Motor Area Template (HMAT). ROI’s for both hemispheres included the following: Primary motor area (M1), primary somatosensory area (S1), dorsal premotor cortex (PMd), ventral premotor cortex (PMv), supplementary motor area (SMA) and pre-supplementary motor area (pre-SMA). An additional ROI was the union of all HMAT masks into a single large mask (union HMAT). Table 2. Report of the top 3 regional accuracies within the Human Motor Area Template (HMAT), as well as from a union of all HMAT regions, for an SVM trained to differentiate wrist extension and flexion using spatially normalized images (realigned, slice-time corrected, and transformed to MNI coordinates) evaluated using 10-fold cross validation.

Fig 4 reports the multivariate classification accuracy, sensitivity, and specificity across all HMAT regions, including the union of HMAT regions. Accuracy, sensitivity, and specificity track closely across the regions of interest.

#### Native acquisition image fMRI input

To investigate whether multivariate classifier model performance could be improved by using a participant-dependent classifier, we performed the same multivariate analysis above in each participant’s native image acquisition space. We created participant-specific region masks based on BA3, BA4, and BA6 utilizing FreeSurfer. The participant-specific BA4 masks were further subdivided into participant- and hemisphere-specific BA4 “hand knob” masks using the criteria of Yousry, et al [35]. These masks were intended to provide more precise and anatomically accurate representations of the regions of interest than the Human Motor Area Template applied to brain images transformed into MNI common coordinates. By forgoing this spatial normalization step, a further benefit is less interpolation applied to the fMRI data.

**Fig 5:**
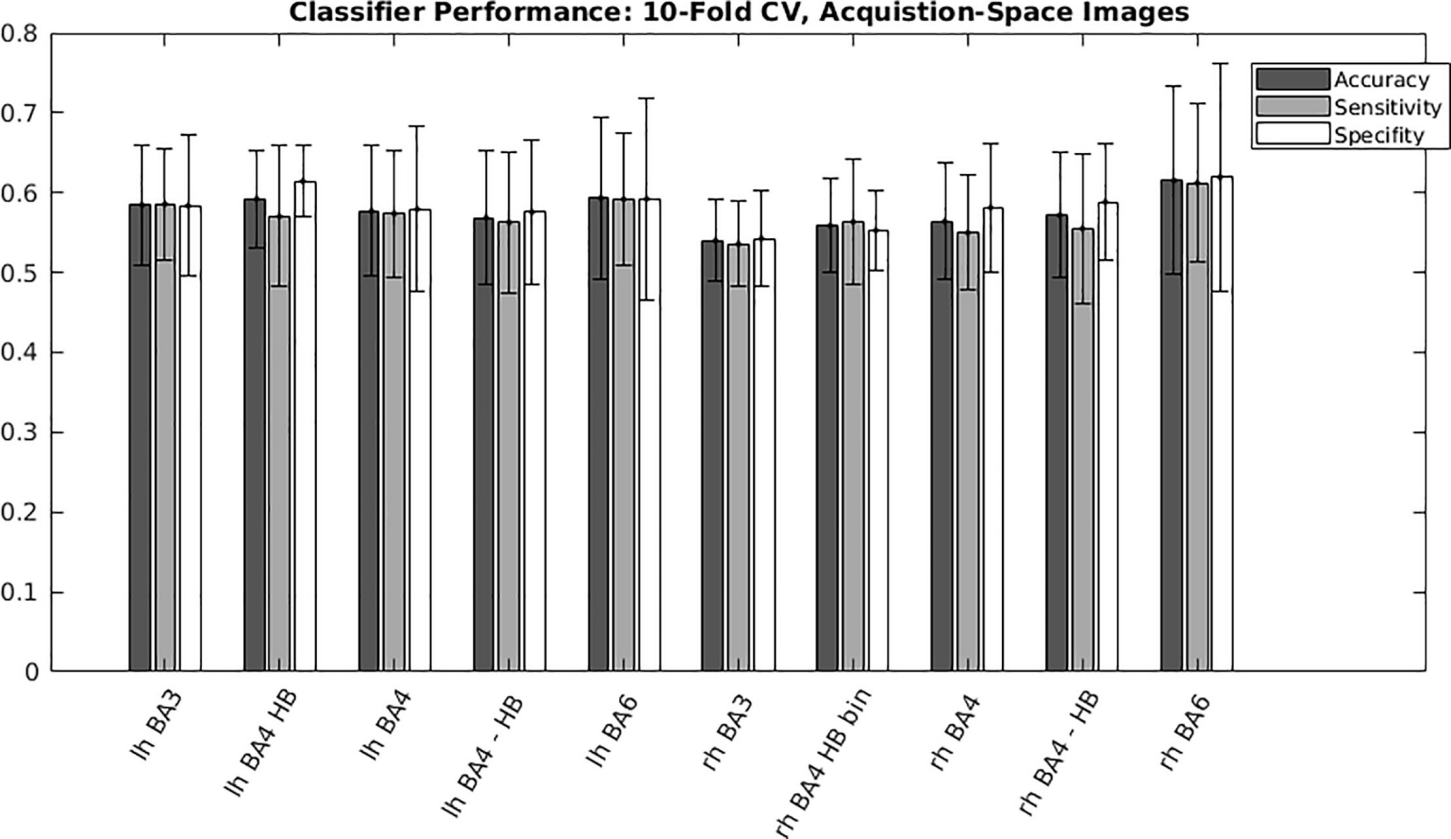
Acquisition-space images: Regional classifier performance in terms of accuracy, sensitivity, and specificity. Brodmann’s areas ROI masks were derived from participant-specific cortical parcellations of native acquisition images, ROIs were investigated in both hemispheres for primary somatosensory cortex (BA3), primary motor cortex (BA4), and pre-motor and supplementary motor areas (BA6). The participant-specific BA4 masks were further subdivided into participant- and hemisphere-specific “hand knob” masks (BA4-HB).

Classification accuracy, sensitivity, and specificity were calculated per participant using 10-fold cross validation (Table 3 and Fig 5). The top 3 regions based on mean accuracy were the right BA6, left BA6, and left “hand knob” with mean accuracies of 61.5%, 59.3%, and 59.2%, respectively. Similar to the spatially normalized images, the accuracy, sensitivity, and specificity track closely across the regions of interest.

**Table 3:** Report of the top 3 regional accuracies from participant-specific hemisphere-specific Brodmann’s areas masks, as well as a union of masks consisting of bilateral BA 3, 4, and 6.

Subject		Accuracy (%)		
	R BA6	L BA6	L HB	BA Union
01	46.6	52.4	55.0	47.0
02	46.7	39.0	50.6	43.0
03	67.7	64.2	70.2	68.6
04	64.5	53.0	57.3	71.0
05	66.6	69.5	57.9	66.0
06	80.6	73.2	57.0	78.2
08	46.1	56.4	56.2	53.0
09	61.2	54.4	60.2	60.2
10	63.3	64.6	58.1	61.8
11	72.1	66.4	69.5	68.1
Mean (SD)	61.5 (11.7)	59.3 (10.2)	59.2 (6.15)	61.7 (11.1)

The SVM was trained to differentiate wrist extension and flexion using acquisition-space images (realigned, slice-time corrected) evaluated using 10-fold cross validation.

### Functional maps of wrist action preference

#### “Winner take all” based on t-statistic, results; third type of analysis

**Fig 6:**
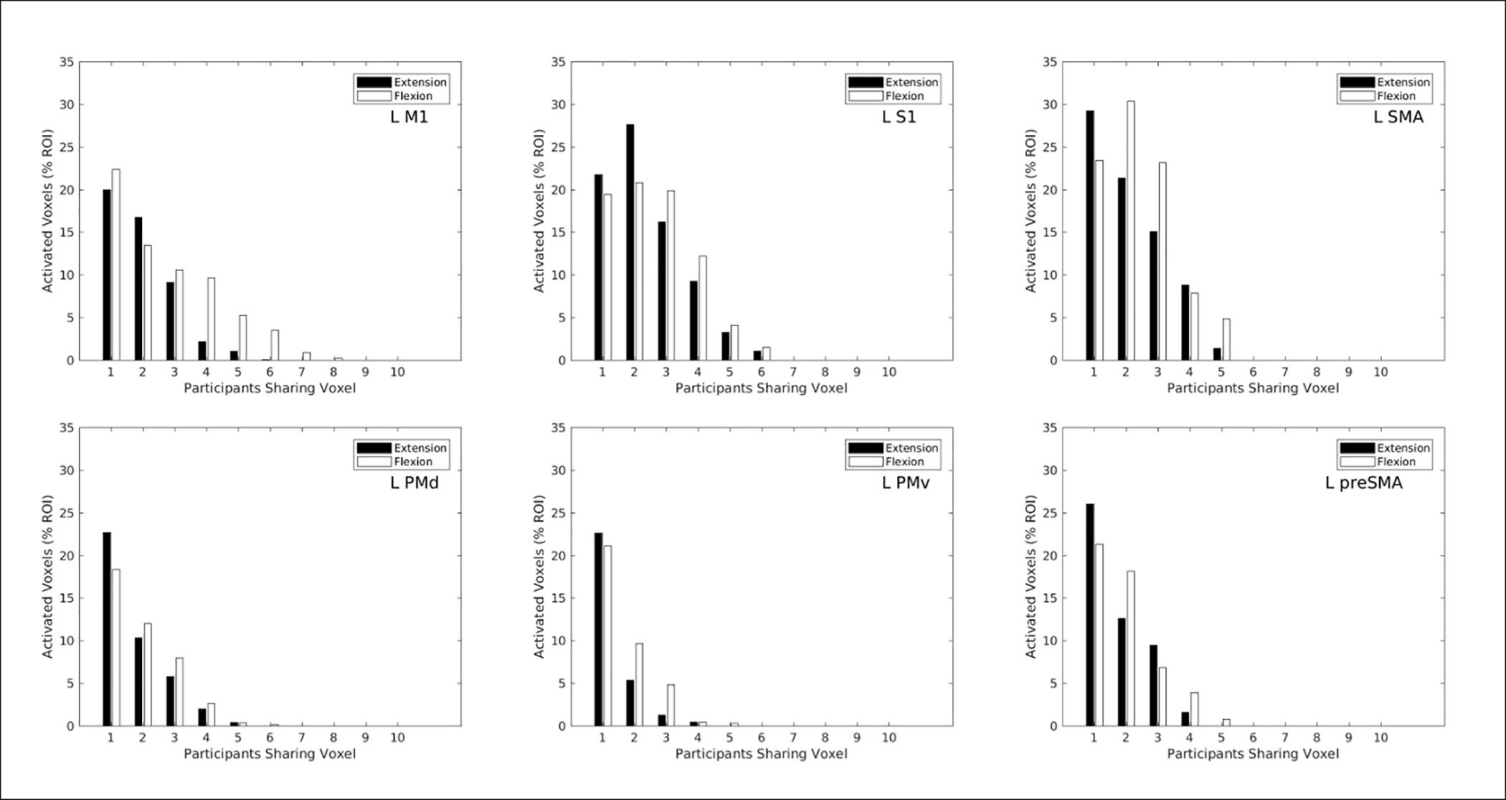
**Histograms comparing wrist extension (black bars) and wrist flexion (white bars) for how many activated voxels (p < 0.001, uncorrected) shared the indicated “winner-take-all” action preference as a function of the number of participants sharing that voxel.** Ordinate height of each bar represents a count of activated voxels (scaled as percentage of all voxels in the selected ROI) that share the same action preference across a given number of participants. Voxels were counted across all participants, using the MNI coordinate system as a common space. The “winner take all” criterion for action preference evaluated which action produced the larger t-statistic in each activated voxel. Different panels present results for the six left hemisphere ROIs from the Human Motor Area Template (top row: left M1, left S1, left SMA; bottom row: left dorsal premotor, left ventral premotor, left pre-SMA).

**Fig 7:**
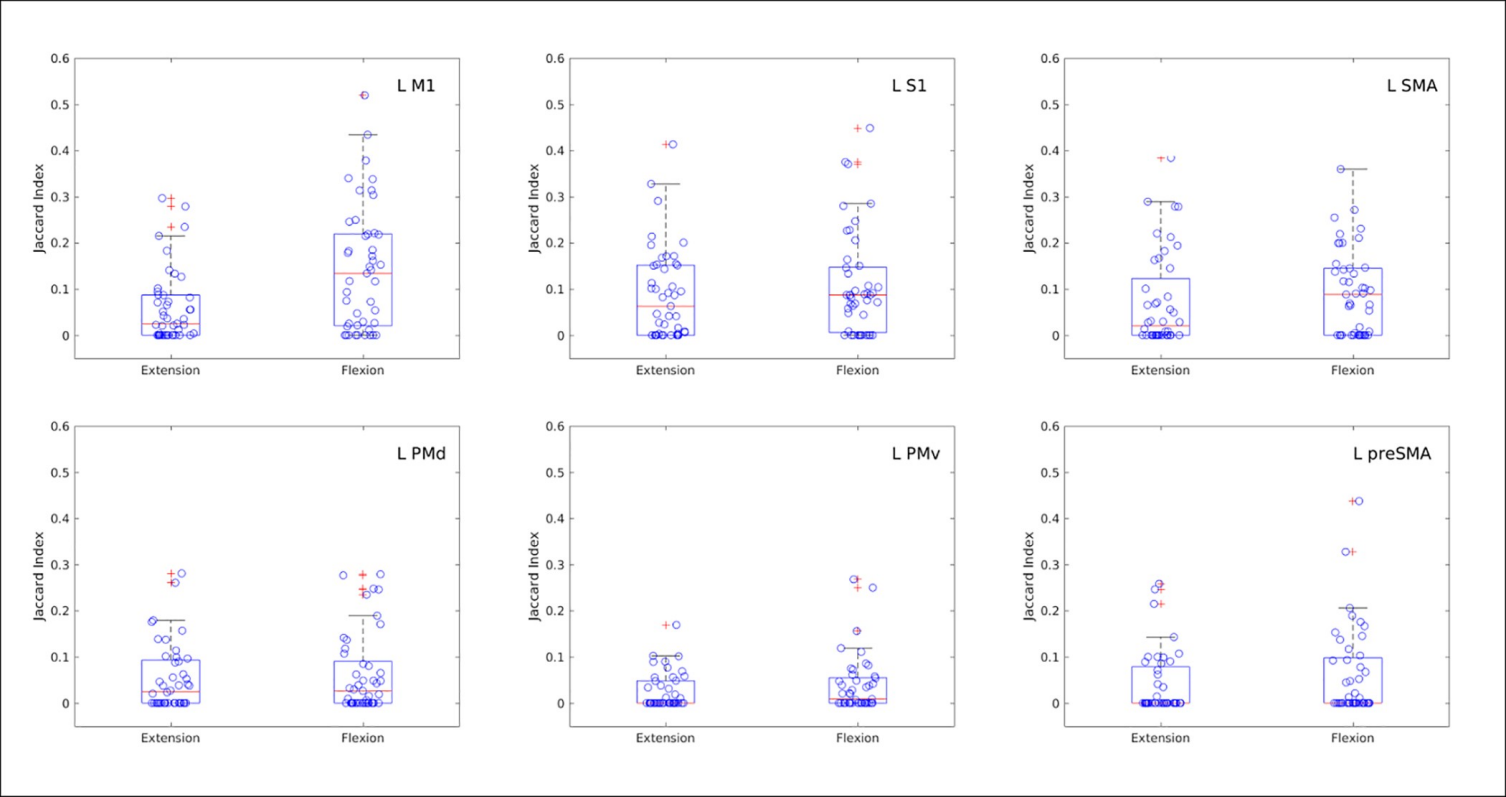
Box plots of the Jaccard index of similarity between pairs of participants’ sets of activated voxels preferring wrist extension or wrist flexion, as evaluated by which action yielded the larger t-statistic. Each Jaccard index of similarity evaluates degree of overlap for two sets of voxel coordinates by calculating the ratio of intersection to union of the sets. For total overlap of two sets, the intersection equals the union so the Jaccard index is 1. Each point represents the index of similarity for voxel sets in MNI common coordinates, derived for the same wrist action, obtained from different participants. All possible pairwise comparisons of the 10 participants are shown. Different panels present results for the six left hemisphere ROIs from the Human Motor Area Template (top row: left M1, left S1, left SMA; bottom row: left dorsal premotor, left ventral premotor, left pre-SMA).

Results of the “winner take all” (WTA) rule for relative magnitude of the t-statistics contrasting action to rest are shown in Figs 6 and 7. This analysis does *not* directly contrast extension versus flexion for significant differences in activation levels. Fig 6 shows histograms comparing relative frequencies of WTA for wrist extension and WTA for flexion presented in separate panels for each left hemisphere region of interest (ROI). Ordinate height of each bar plots how many activated voxels (p < 0 .001 uncorrected) in MNI common space possess the same label across participants, as a function of how many participants shared that label as shown on the abscissa. For ease of comparison across ROIs, the ordinate heights across the six panels of Fig 6 are scaled as proportions of the total number of voxels in each respective ROI. In all six panels, moderate percentages of ROI voxels (19%– 29%) were labeled as extension WTA or as flexion WTA for only 1 out of the possible 10 participants (not the same individual for all voxels). These activated voxels were located inconsistently between subjects, and in five ROIs (not left S1) more such inconsistently located voxels preferred extension than preferred flexion. No voxel in any panel had complete consistency in sharing the same label across all 10 participants. However, the left M1 ROI (upper left panel) exhibited percentages of voxels preferring flexion in 3 to 8 out of the 10 participants which exceeded the percentages of voxels preferring extension. This pattern also exists in the other five ROIs shown, and it indicates a moderately consistent location preference for flexion wrist action.

Fig 7 illustrates box plots of the spatial similarity of both the extension and flexion WTA labels as estimated by the Jaccard similarity coefficient, which is based on the degree of overlap calculated as the ratio of intersection to union of the label sets. Each plotted point in Fig 7 represents the similarity coefficient of two label maps in MNI common space derived for the same wrist action but from different participants. The higher the similarity, the closer the Jaccard coefficient is to 1. Regions with relatively high similarity coefficients include the left M1 (flexion), left S1 (flexion and extension), and left SMA (flexion). Fig 7 compliments the histograms presented in Fig 6 by providing information about the spatial arrangement of different participants’ maps for both the extension and flexion conditions. By revealing a relatively high degree of similarity for the spatial arrangement of flexion labels in the left M1, Fig 7 supports the notion that left M1 location preferentially codes for wrist flexion. In addition, the left S1 region appears to have location consistency (up to 6 out of 10 participants) preferentially coding for flexion and for extension.

#### Action preference (wrist extension vs flexion) based on percent signal change (movement—rest), results; fourth type of analysis

Huber et. al., used the difference in brain signal for two different movement actions to determine a region’s preference for one condition relative to the other [48]. The variable that they used was percent signal change (movement–rest) for each of the movement tasks. We performed similar calculations.

**Fig 8:**
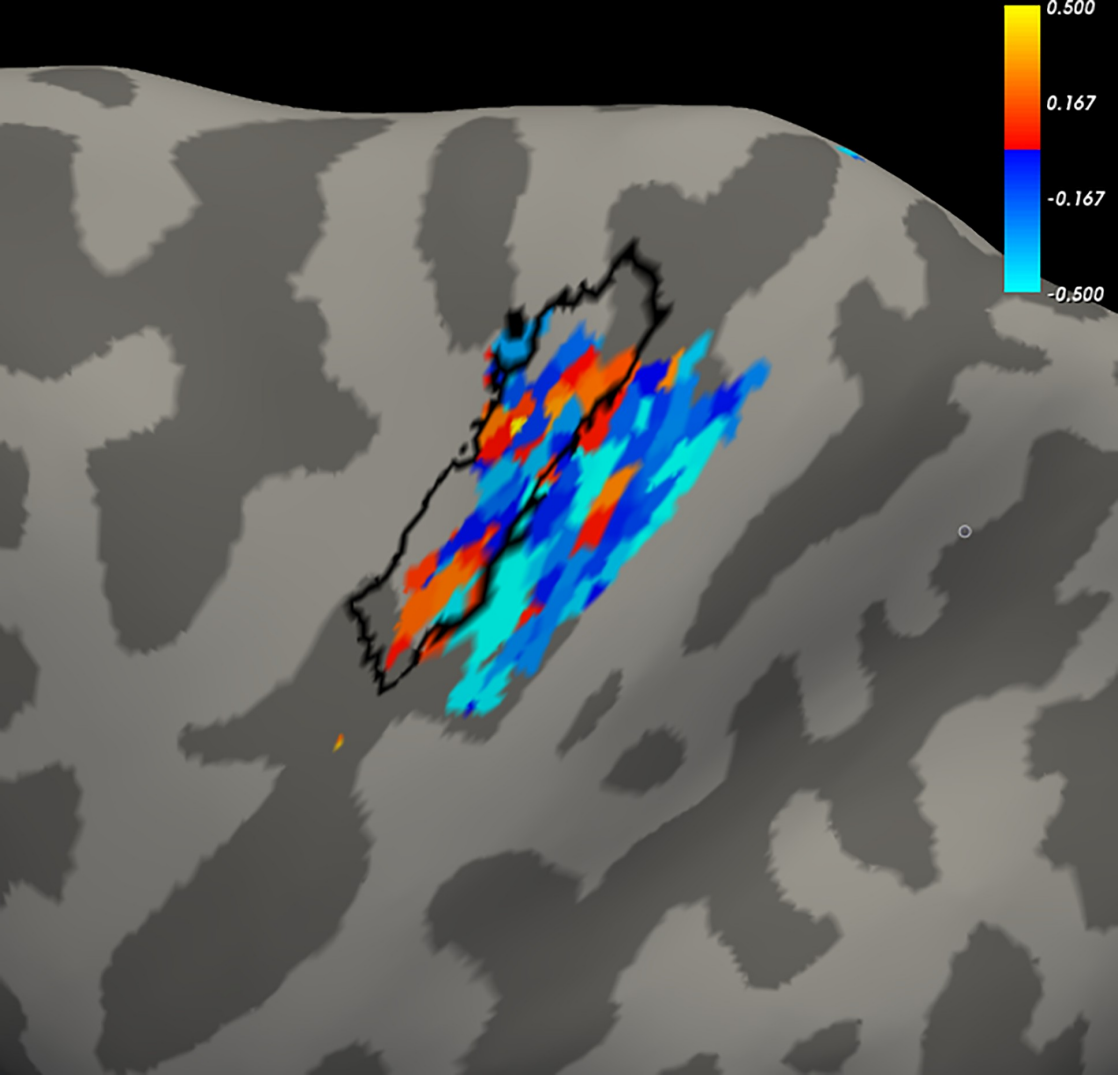
Subject 1 example heat map of wrist action preference (remaining individual subject data are located in the S1 File). Surface projections of native acquisition images show left primary motor (L BA4ap) and left primary somatosensory (L BA3ab) to illustrate co-localization of voxel preferences for extension (yellow/red) and flexion (blue/aqua), as determined by the difference in percent signal change from rest. The cortical surface of the hand knob is outlined in black. Functional percent signal change was calculated for all voxel time courses. The wrist extension reference was calculated as 100 X (extension signal—baseline)/baseline; and the flexion reference was 100 X (flexion signal—baseline)/baseline. The extension and flexion signals were calculated as the mean voxel time course over the movement condition (averaged across all trials). The baseline values were calculated using the median value of the preceding and the subsequent rest periods, which were then averaged, to minimize effects of residual drift. Difference between extension and flexion was calculated for each run. Voxels were filtered using a union of t-maps for ’wrist extension versus rest’ and ’wrist flexion versus rest’ (i.e., active voxels for wrist extension, wrist flexion, or both; t threshold, p < 0.001 uncorrected, such that only suprathreshold activated voxels were considered for further analysis. Session average was the mean of the derived extension-flexion percent signal change across runs within the session.

For a given participant, the average of the difference between extension and flexion was calculated, according to the variable of percent signal change during extension (extension–rest) or flexion (flexion minus rest). Fig 8 displays an example, for Subject 1, in the form of a heat map for activated voxels, t-statistic threshold p < 0.001 (uncorrected) (Data for the remaining subjects are located in the S1 File, Fig 8). The heat map is projected onto the left hemisphere cortical surface for the BA4 (primary motor) and BA3 (primary somatosensory) ROIs. For reference, we included a black outline of each participant’s primary motor “hand knob” to help orient the reader to the cortical surface view. Although we cannot pinpoint a unifying feature across participants, moderately non-random patterns of warm and cool colors in each participant’s heat map may suggest spatial organization oriented to extension- or flexion-related activations, respectively. Both the motor and somatosensory ROIs generally co-localize for wrist flexion preference (cool colors of blue/aqua). Somatosensory ROIs seem to co-localize for wrist extension preference (warm colors yellow/red) in participants C02, C04, C05, C08, and C09; motor ROIs apparently do so in participants C01 and C03.

Run-to-run reproducibility of these heat maps was assessed using within-session runs in each participant’s native image acquisition space. Each activated voxel (p < 0.001 uncorrected) was assigned a label based on the sign of the difference between extension percent signal change and flexion percent signal change. Voxels exhibiting a greater percent signal change from rest during extension (versus change from rest during flexion) were assigned a label value of 1, and 0 was assigned as label value otherwise (mutually exclusive outcomes). Frequency estimates for the between-runs extension preference consistency of each voxel were calculated as the voxel-wise sum across runs of its labels [0,1]. This sum ranged from 0, which indicated consistent flexion preference throughout, to the total number of runs (which varied by participant), which indicated consistent extension preference throughout.

**Fig 9:**
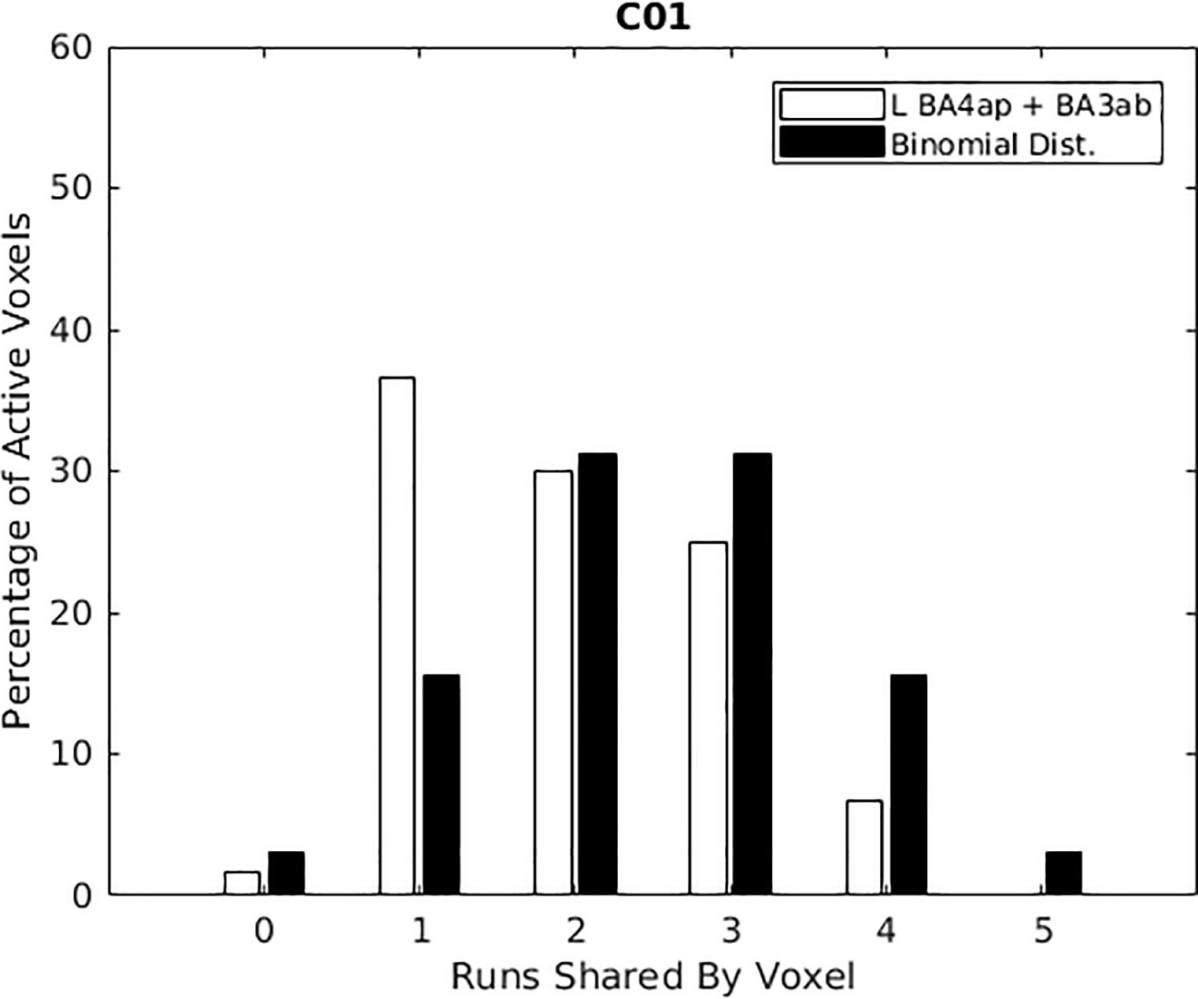
Histograms for left primary motor and somatosensory cortices combined. Ordinate height of the white bars represents a count of activated voxels (p < 0 .001 uncorrected; scaled as percentage of all voxels in the selected ROIs) that showed larger percent signal change during wrist extension than during wrist flexion. These counts are plotted as a function of the number of within-session data collection runs for which extension action preference was exhibited. Black bars show for comparison the corresponding binomial distributions that would be expected given randomly assigned mutually exclusive preference labels if no true preference for extension or flexion existed (i.e., the null hypothesis). Different panels present results for the 10 participants, ordered by participant ID left to right and top to bottom. Note a trend for white bars to be taller than black bars in the left half of the plots, white bars to be shorter than black bars in the right half of the plots. For five participants (C01, C02, C03, C04, C11), observed frequencies differed significantly from binomial distributions (Kolmogorov-Smirnov tests, p < 0.05).

An example histogram is, shown in Fig 9 for Subject 1 (all other individual subject data are located in the S2 File, Fig 9). Fig 9 shows data for both the left primary motor cortex (L BA4ap) and the left primary somatosensory cortex (BA3ab). Ordinate height of the white bars shows the percentage of voxels in these two ROIs that showed greater activity during wrist extension than during wrist flexion (p < 0 .001 uncorrected). This information is shown in Fig 9 for Subject 1, for each of the runs during data acquisition, with each run listed on the abscissa of Fig 9. Black bars show for comparison the corresponding binomial distributions that would be expected given randomly assigned mutually exclusive preference labels if no true preference for extension or flexion existed (i.e., the null hypothesis). The observed data for participants C01, C02, C03, C04, C09, C10, and C11 appear to have more voxels showing extension preference in only a few runs (white bar taller than the associated black bar). Observed frequencies differed significantly from binomial distributions (p < 0.05, Kolmogorov-Smirnov tests) for C01, C02, C03, C04, and C11.

These within-subject action preferences for activations obtained from native image acquisitions with percent signal change comparisons are congruent with the between-subjects “winner take all” analysis based on MNI common coordinate transformations and t-statistic comparisons. Both analyses show more consistent flexion preference than extension preference. Color concentrations on the within-session heat maps (Fig 8) are qualitatively consistent with the between-runs histograms of Fig 9 inasmuch as flexion preferences (cool colors) are more evident on the cortical surface maps and more consistent across runs for most participants. Larger mean activation volumes across participants for flexion versus rest than for extension versus rest were found (Table 2). Imbalances in these features of cortical activations favor flexion over extension.

## Discussion

These results contribute to the literature in three ways. First, to our knowledge, this is the first study to directly investigate fMRI activations inferred from the hemodynamic response patterns of cortical tissue while wrist flexion and wrist extension were being performed in an active movement paradigm, i.e. an movement opposed only by gravity, changing muscle length and joint angle. Second, this study is the first to report on the capability of Support Vector Machines (SVM) to discriminate patterns of brain activity associated with two functional movements performed about the same joint, and to discuss its potential for use in a real-time neurofeedback paradigm aimed at rehabilitation. Third, this study reports results and detailed methods for several different signal processing methods for characterizing brain signals for single joint wrist flexion and extension movements.

We explored the capability of four different signal processing methods to differentiate fMRI signals during healthy adult wrist extension versus wrist flexion in the same limb, inasmuch as such knowledge could provide an important step towards building a neurorehabilitation tool for restoring coordinated wrist movements in stroke survivors who are unable to differentially control those two movements. A number of findings reflect the difficulty in differentiating the neural control for these two wrist movements. In healthy adults, we found high individual variability in brain pattern activation for both wrist extension and wrist flexion. In brain maps for any given individual, voxels could readily be identified in motor cortices which behaved significantly differently during wrist extension versus rest or during wrist flexion versus rest. In most participants, we observed flexion-related activation volumes exceeded extension-related activation volumes, but a greater number of voxels were common between these two wrist actions. And to further complicate matters, the unique voxels were closely adjacent to voxels that were common to both wrist movements, and further, the uniquely active voxels appeared to be of much lesser volume versus the voxels that were common to both wrist and extension movements. These findings may explain the difficulty in producing high accuracy and sensitivity in the group analyses and in the classification methods.

### Univariate group-level analysis

We used the widely accepted methods of univariate analysis for fMRI activations, specifically, application of the general linear model to generate statistical parametric maps reflecting hemodynamic time courses. Voxels could readily be identified in motor cortices which behaved significantly differently during wrist extension versus rest, or behaved significantly differently during wrist flexion versus rest. On average, flexion-related activation volumes exceeded extension-related activation volumes. For some participants, some voxels in some ROIs were activated exclusively by extension but not by flexion (or vice versa). However, in group-level analysis, our methods were not able to identify unique classes of voxels which behaved significantly differently during wrist extension than during wrist flexion. We are confident that these different wrist actions are mechanistically related to different neural activities of some kind but limitations on our ability to capture and analyze those activities, perhaps the relatively large size of the present functional voxels, hinder identifying them fully.

### Multivariate analysis

This study was able to classify fMRI activity associated with wrist extension and wrist flexion using multivariate analysis via Support Vector Machine (SVM); however, the performance metrics of accuracy, sensitivity, and specificity were modest. Modest classification accuracy presents challenges to adopting multivariate SVM classification for use in a real-time neurofeedback rehabilitation paradigm.

Yoshimura et al. [31] studied fMRI activations during isometric wrist extensions or flexions made from pronated or suppinated postures of the constrained forearm. The isometric contractions did not change joint angle or muscle length whether directed up or down with respect to gravity. The isometric task demands force production by the muscle. Activations in their study could be classified using multivariate techniques into intrinsic versus extrinsic motion coordinate frames. Our greater difficulty in classification of extension versus flexion could be due to our isotonic movement task (simple active range of motion against no resistance (small force production) except that of gravity). That is, the wrist was not constrained kinematically, and the only possibly negligible force was gravity, during a simple, almost automatically performed movement. Thus, the greater force production in the Yoshimura study could have demanded greater unique volume of activation for their tasks.

### Functional maps of wrist action preference

We saw preferential activation favoring extension more than flexion in some voxels, and vice versa in other voxels. Heat maps showed evidence of moderate co-localization of those voxels favoring flexion and to a lesser degree, co-localization of voxels favoring extension. Patterns of co-localization differ between participants and appear to be temporally variable within participants, and thus seem challenging to adopt for use in a real-time neurofeedback rehabilitation paradigm.

### General considerations

No fMRI signature was identified as able to be reliably discriminated from comparison measures by any of the four different signal processing methods used. Therefore, the receiver operator characteristics (ROC) would each be expected to be along the line defined by lack of discrimination. In such cases, the true positive rate (TPR) would equal the false positive rate (FPR = TPR), and area under the curve (AUC) would be 0.5.

Even though good brain pattern differentiation has not been accomplished for such tasks as wrist extension versus wrist flexion or other abnormally co-activated movements, there has still been some success in developing and/or using neural feedback BCI systems to treatment motor dysfunction after stroke [e.g., 10,21,49]. A more sophisticated BCI system with ability to differentiate spatially uniquely activated voxels (with fMRI) might further improve BCI results, but these new discoveries have yet to be solidified and documented. If these discoveries are realized, the differentiated signal could be provided to the stroke survivor user the means to mitigate the abnormally elevated antagonist muscle activity and elevate the abnormally minimal agonist muscle activity. This current study on healthy adults is a cautionary step forward in that it provides a description of four methods that did not prove useful in differentiation of the needed motor tasks of wrist extension and wrist flexion. Nevertheless, differentiation and generation of feedback signal would be very useful for stroke survivors; additionally, the current results point to what may be necessary for future successful methods.

### Limitations and future directions

Neural activity is addressed indirectly by fMRI signals that capture hemodynamic responses by way of blood oxygenation level magnetic contrast. One caveat is that the metabolic demand evoking the hemodynamic response does not identify whether the underlying synaptic activity creating that demand had exitatory or inhibitory neural effects. A second caveat is that the fMRI signal is volume-averaged over the extent of each spatially encoded voxel containing a large population of neurons, blurring out fine spatial details, and time-averaged between excitation pulses, blurring out fine temporal details. With our 3T instrument we obtained fMRI signals in voxels of 3x3x3 mm at TR = 3 sec and recognize those resolution limits. Limitations of the study design were small sample size and limited reliability testing, insufficient resources for deconvolution-based secondary fMRI analyses, plus the lack of kinematic measurements characterizing the wrist movements. That is, we did not directly measure kinematic properties of wrist flexion-extension, radial-ulnar deviation, or wrist rotation. There could have been undetected variability among the movements executed despite identical instructions in terms of movement path, speed, joint angle, and range of motion. These undetected differences may have contributed to the observed variability. However, the movement was a simple movement performed many times daily, without thought, which is the movement we wished to study. That is, the purpose of the analysis was not to test a kinematically constrained movement of some force, but to study the simple active wrist extension movement that is performed many times daily in preparation for grasp. Sample size of 10 could have contributed to the result of our group analyses showing no statistically significant flexion-extension differences. Given the observed individual variability of activation maps (and the voxel size in the current study), it is possible that adding more subjects to our data would not necessarily yield a more representative mean activation, and would not necessarily or substantially improve discrimination between wrist movement types. This realization provided part of the rationale for including the discriminatory analyses methods of Huber et al. [48] and Meier et al. [47]. Also, we can note that this situation argues for the application of precision (that is, ‘custom’) medicine that is individualized specifically for the individual. Precision medicine is a rapidly emerging concept and should be considered in neurorehabilitation and neural feedback methods. Precision neurorehabilitation (individually customized) and neural feedback of brain signal for a given stroke survivor could include the use of a brain signal map generated from his/her own non-lesioned hemisphere controlling the unimpaired limb; however, that concept inherently includes the potential confounds of disruptions of neural networks in the non-lesioned hemisphere caused by the stroke, as well as the normally occurring asymmetry of right and left, brain hemispheres. Finally, we were able to conduct the four methods of analysis presented here, with constraints on time and resources limiting further work. Other methods of data acquisition and analyses could potentially yield a better differentiation of wrist extension versus wrist flexion. In contrast to the block design utilized in the current study, one possibility is acquiring brain signal using an event-triggered design, in which each motor movement is acquired separately, and analyzing the data from the event-triggered acquisition. Comparing the brain signal for the two tasks in this manner could be a major advantage.

The results indicate that future directions of inquiry are required, if a viable brain neurofeedback system is to be developed for neurorehabilitation. These could include methods that are currently emerging or yet to be developed. Some possibilities include the following: invasive acquisition of brain signal; new non-invasive methods for acquiring brain signal; or more precise existing methods of data acquisition including a 7T machine, smaller voxel size, and/or single task, event-triggered data acquisition and analysis.

## Conclusions

We explored the capability of four different signal processing methods to differentiate fMRI signals during healthy adult wrist flexion versus wrist extension in the same limb, inasmuch as such knowledge could provide an important step towards building a neurorehabilitation tool for restoring coordinated wrist movements in stroke survivors. We encountered a number of obstacles to obtaining clear group results in healthy adults. These obstacles included the following: high variability across healthy adults in all measures studied; close proximity of uniquely active voxels to voxels that were common to both the extension and flexion movements; in general, higher magnitude of signal for the voxels common to both movements versus the magnitude of any given uniquely active voxel for one type of movement. Our results indicate that greater precision in imaging will be required to develop a truly effective neural feedback system. A future effective neurofeedback system will provide the individually-based neurorehabilitation neural feedback signal that is necessary for recovery of motor control after stroke, given the uniqueness of brain networks across individuals. It is not yet clear how or if a more sophisticated neural feedback signal for such a system will be possible. Future studies will answer that question.

## Supporting information

S1 File Individual subject data for Fig 8. The S1 File Supporting Information provides data for each subject separately.
(DOCX)

S2 File Individual subject data for Fig 9. The S2 File Supporting Information provides data for each subject separately.
(DOCX)
