# Supplementary material for: Four methods of brain pattern analyses of fMRI signals associated with wrist extension versus wrist flexion studied for potential use in future motor learning BCI
Source: PLoS One. 2021 Aug 17;16(8):e0254338. doi: 10.1371/journal.pone.0254338 (PMC8370644; doi:10.1371/journal.pone.0254338)

**Figure 8. Individual Heat Map of Wrist Action Preference Based on Percent Signal Change (Action versus Movement). Brain maps are shown for each of 10 healthy adults performing simple wrist extension (Figures 8.a. through 8.j.)**

Surface projections of native acquisition images showing left primary motor cortex (L BA4ap) and left primary somatosensory cortex (L BA3ab) to illustrate co-localization of voxel preferences for extension (warm colors, yellow/red) and flexion (cool colors, blue/aqua), as determined by magnitudes of the difference in percent signal change from rest that was evoked by those actions, respectively. Averages across runs are shown for activated voxels, t-statistic threshold, p < 0.001 uncorrected. Outlines of each participant’s primary motor cortex “hand knob” are shown in black to help orient the reader to the cortical surfaces depicted. (Subject 07 was omitted from analysis, as described in methods).

Fig 8.a. Subject C01


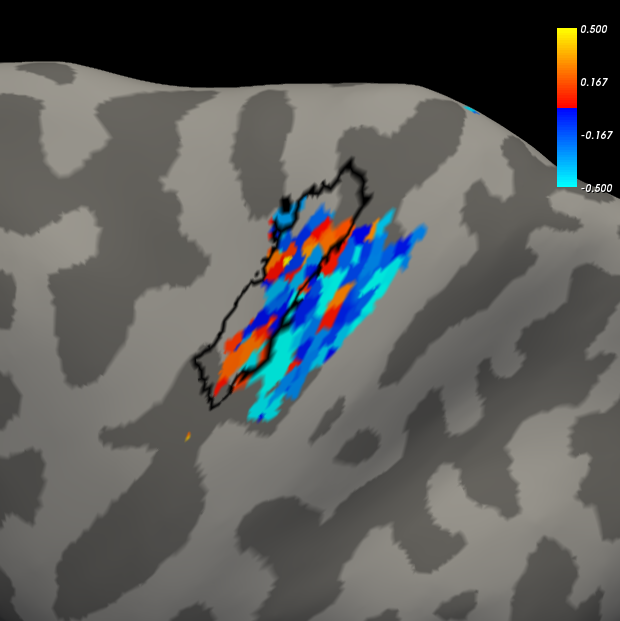


Fig 8.b. Subject C02


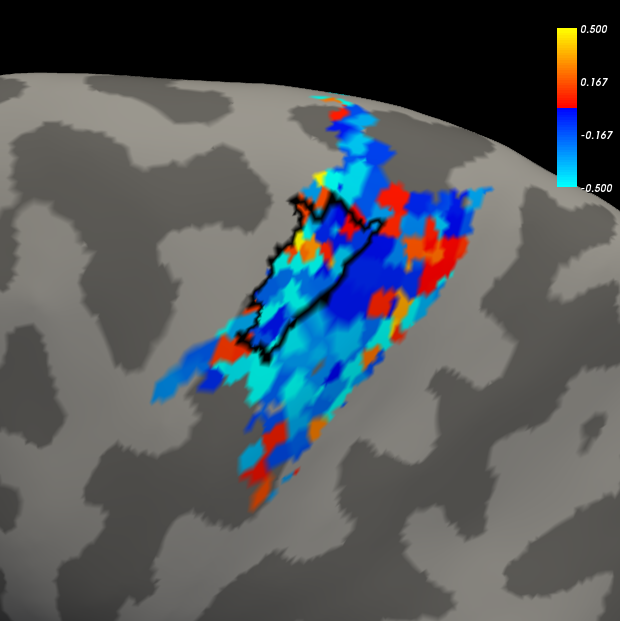


Fig 8.c. Subject C03


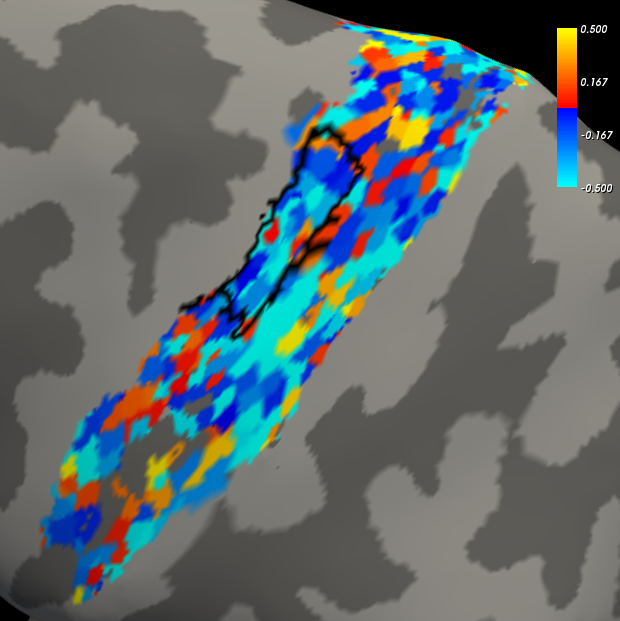


Fig 8.d. Subject C04


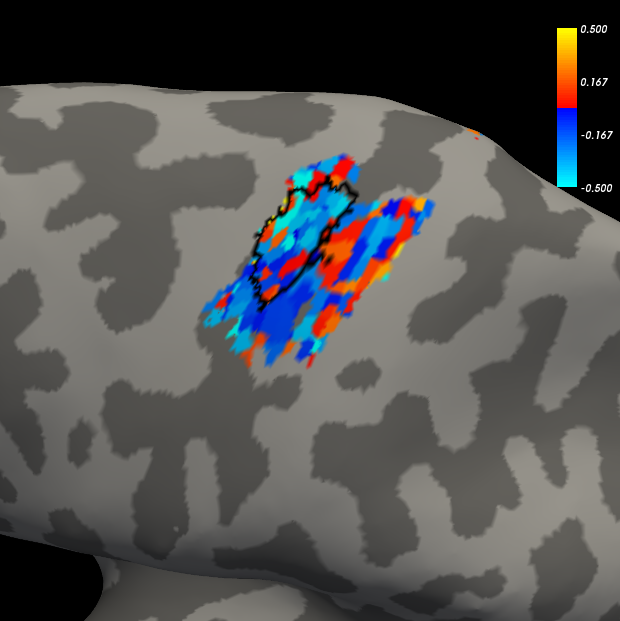


Fig 8.e. Subject C05


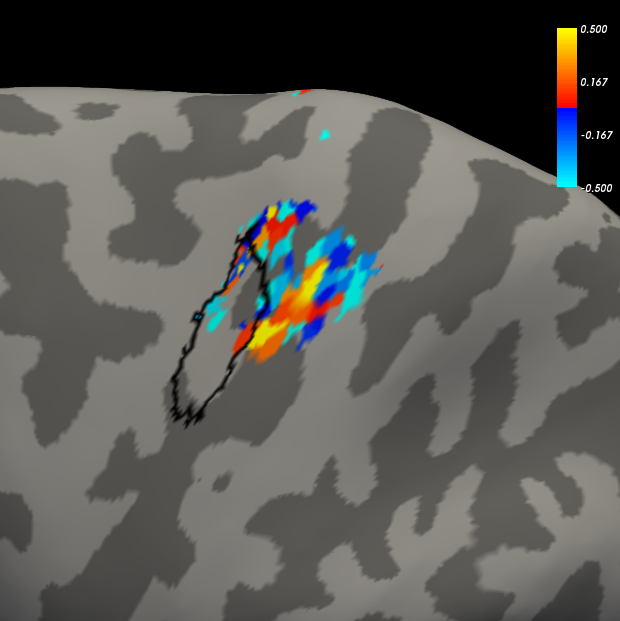


Fig 8.f. Subject C06


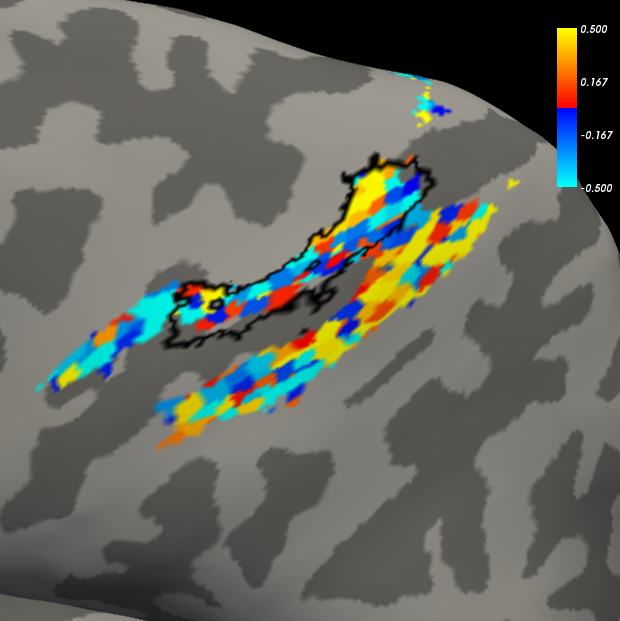


Fig 8.g. Subject C08


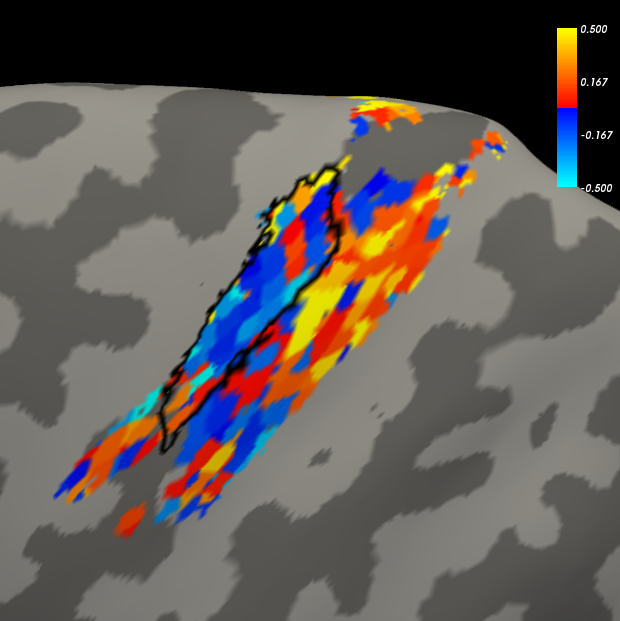


Fig 8.h. Subject C09


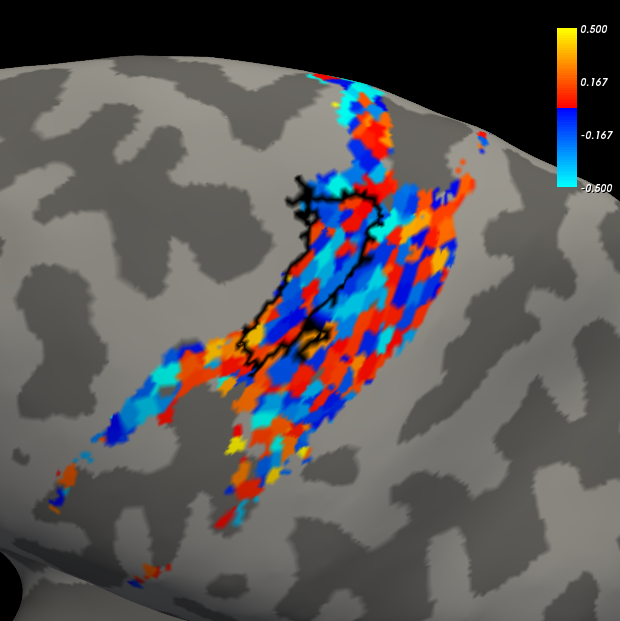


Fig 8.i. Subject C10


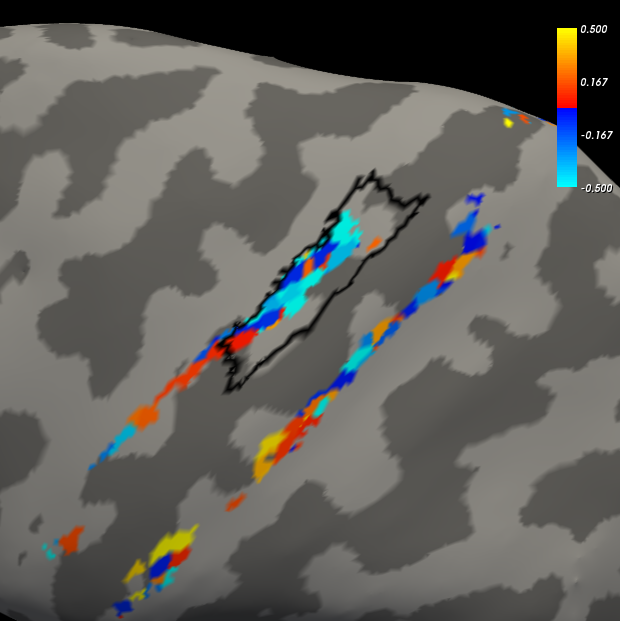


Fig 8.j. Subject C11


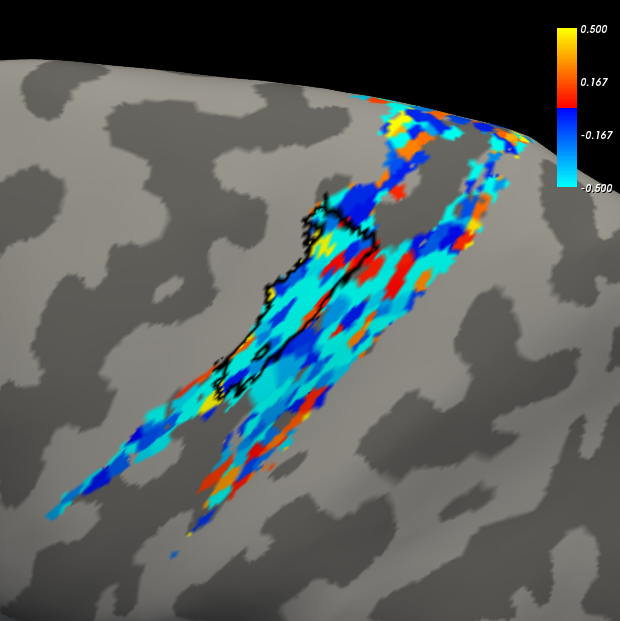

Supplement: S1 File — The S1 File Supporting Information provides data for each subject separately. (DOCX) [file pone.0254338.s001.docx]
