# Supplementary material for: Four methods of brain pattern analyses of fMRI signals associated with wrist extension versus wrist flexion studied for potential use in future motor learning BCI
Source: PLoS One. 2021 Aug 17;16(8):e0254338. doi: 10.1371/journal.pone.0254338 (PMC8370644; doi:10.1371/journal.pone.0254338)

**Fig 9. Histograms for left primary motor and somatosensory cortices combined. Data for each of 10 subjects are provided in separate panels below (Figures 9.a. through 9.j.) .**

Ordinate height of white bars represents a count of activated voxels (p < 0 .001 uncorrected; scaled as percentage of all voxels in the selected ROIs) that showed larger percent signal change during wrist extension than during wrist flexion. These counts are plotted as a function of the number of data collection runs for which extension action preference was exhibited. Black bars show, for comparison, the corresponding binomial distributions that would be expected given randomly assigned mutually exclusive preference labels if there were no true preference for extension or flexion (i.e., the null hypothesis). We note a trend for white bars to be taller than black bars for some participants, and for white bars to be shorter than black bars. For five participants (C01, C02, C03, C04, C11), observed frequencies differed significantly from binomial distributions (Kolmogorov-Smirnov tests, p < 0.05).

**Fig 9.a. Subject C01**
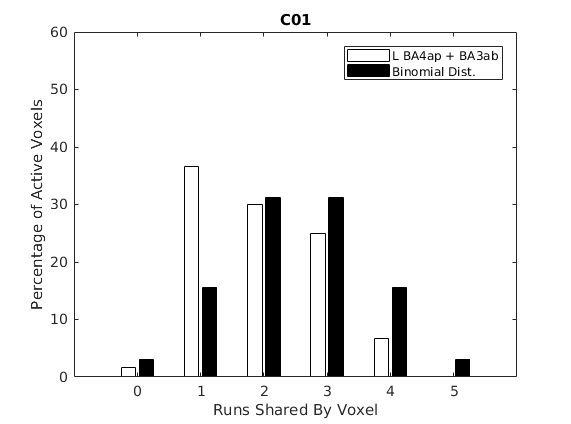


**Fig 9.b. Subject C02**


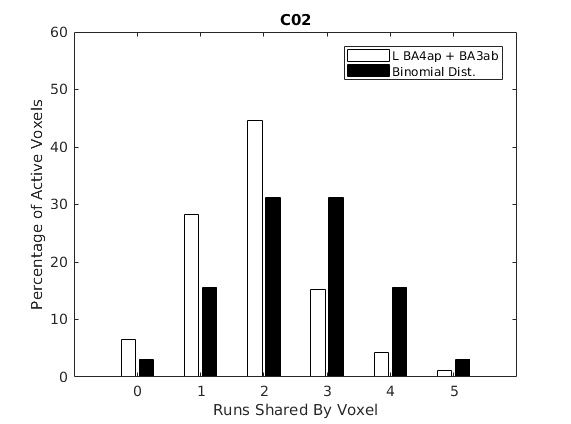


**Fig 9.c. Subject C03**


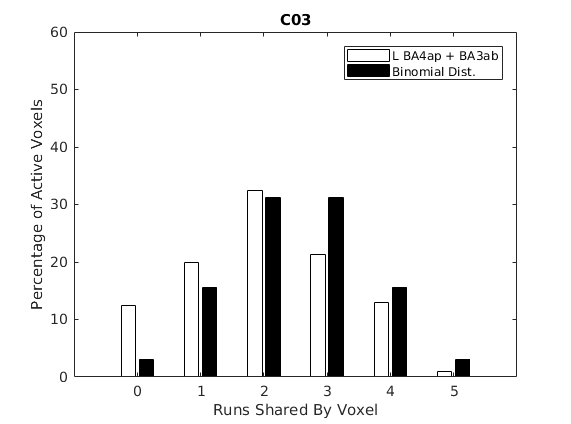


**Fig 9.d. Subject C04**


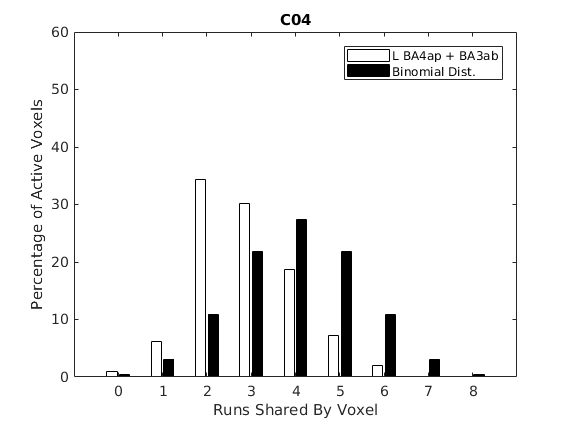


**Fig 9.e. Subject C05**


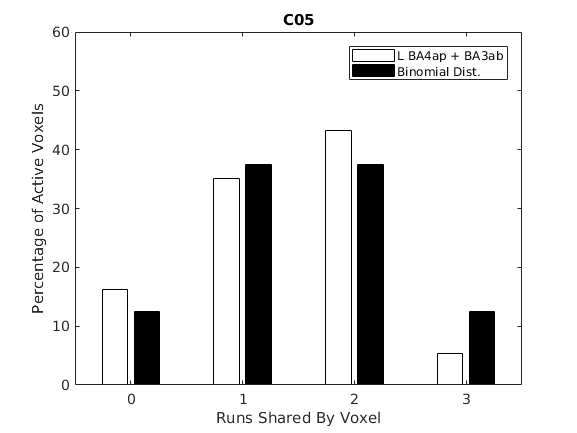


**Fig 9.f. Subject C06**


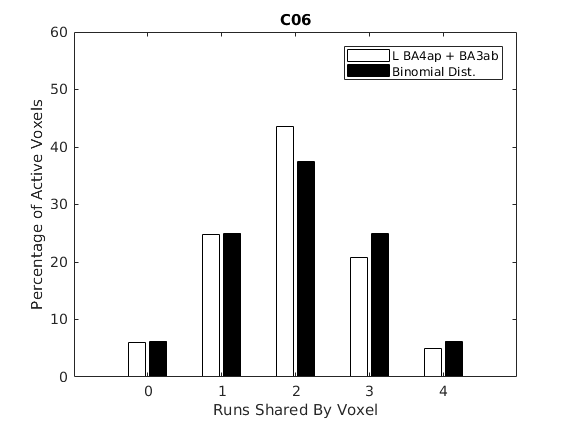


**Fig 9.g. Subject C08**


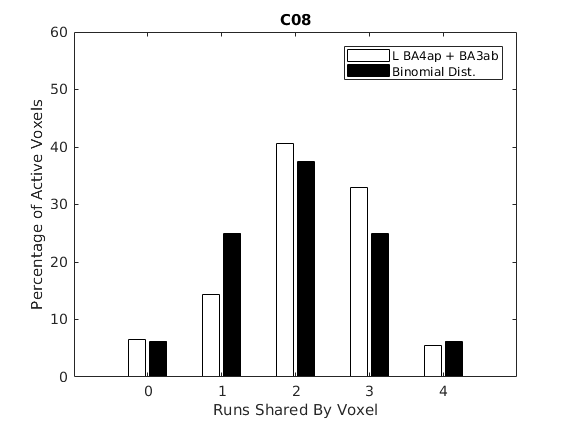


**Fig 9.h. Subject C09**


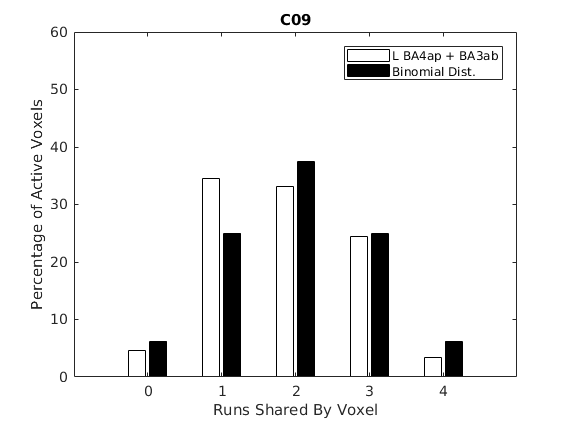


**Fig 9.i. Subject C10**


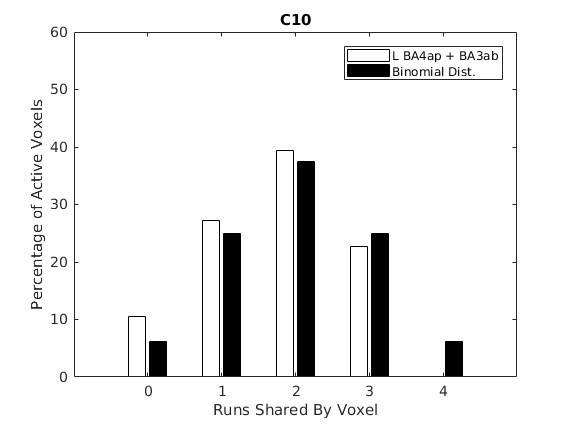


**Fig 9.ja. Subject C11**


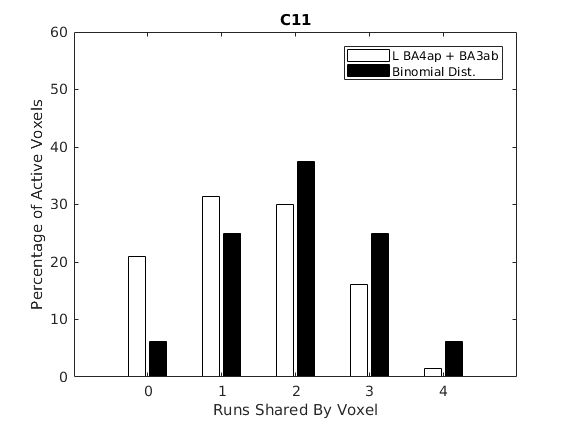

Supplement: S2 File — The S2 File Supporting Information provides data for each subject separately. (DOCX) [file pone.0254338.s002.docx]
